# Improving photovoltaic water pumping system performance with ANN-based direct torque control using real-time simulation

**DOI:** 10.1038/s41598-025-88330-8

**Published:** 2025-02-02

**Authors:** Ikram Saady, Btissam Majout, Ismail El Kafazi, Mohammed Karim, Badre Bossoufi, Najib El Ouanjli, Said Mahfoud, Ahmed Althobaiti, Thamer A. H. Alghamdi, Mohammed Alenezi

**Affiliations:** 1 Laboratory of Engineering Modelling and Systems Analysis, Sidi Mohamed Ben Abdellah University, Fez, Morocco; 2 SmartiLAB EMSI-Rabat, Honoris United Universities, Rabat, Morocco; 3 Higher School of Technology, Moulay Ismail University, Mekness, Morocco; 4 Industrial Technologies and Services Laboratory, Higher School of Technology, Sidi Mohamed Ben Abdellah University, Fez, Morocco; 5 Department of Electrical Engineering, College of Engineering, Taif University, P.O. Box 11099, Taif City, 21974 Saudi Arabia; 6 Wolfson Centre for Magnetics, School of Engineering, Cardiff University, Cardiff, CF24 3AA UK; 7 Electrical Engineering Department, Faculty of Engineering, Al-Baha University, Al-Baha, 65779 Saudi Arabia

**Keywords:** Photovoltaic water pumping system, Artificial neural networks, Direct torque control, Induction motor, dSPACE DS1104, Electrical and electronic engineering, Physics

## Abstract

Photovoltaic Water Pumping Systems (PVWPS) have become increasingly important as a renewable energy solution in rural areas, providing energy independence, cost savings, and environmental friendliness. This system has two main controllers. The first controller is employed to maximize power extraction from the PV array by controlling the duty ratio of the DC-DC boost converter. The second controller is responsible for regulating the operation of the induction motor through the switching pulses of the Voltage Source Inverter (VSI). These two controllers play an essential role in the system, which increases efficiency and performance. Therefore, the innovative aspect of this work consists of introducing Artificial Neural Networks (ANNs) based on each PVWPS controller. On the one hand, ANN-based MPPT is implemented to ensure optimal performance of the PV array under varying irradiation levels. On the other hand, to overcome the defects and problems caused by Direct Torque Control (DTC), such as flux and torque ripples, high switching frequency, and challenges at low speeds, an ANN-based DTC is proposed in which each of the hysteresis comparators, switching table, and speed controller in the DTC are replaced by ANN controllers. The PVWPS based on the proposed controls is thoroughly modeled and simulated using MATLAB/Simulink software and validated using dSPACE DS1104 Board. The results demonstrate significant improvements, including a 75.51% reduction in flux ripples, a 77.5% reduction in torque ripples, a 44.79% improvement in response time, and an increase in the water quantity. Furthermore, the Real-Time simulation and visualization obtained are consistent with the simulation outcomes.

**Supplementary Information:**

The online version contains supplementary material available at 10.1038/s41598-025-88330-8.

## Introduction

Recently, the increasing demand for Renewable Energy (RE) has received significant attention from many countries worldwide due to its environmental advantages, reducing greenhouse gas emissions and decreasing reliance on fossil fuels, thus ensuring a suitable future for human life, and meeting human needs^1^. RE sources include various categories, such as wind, solar, hydroelectric, and geothermal energy^2^. Among these, Solar Energy (SE) is particularly noteworthy due to its ease of harnessing, natural availability, and noise-free operation. SE offers significant benefits in rural areas, particularly where electricity is unavailable due to remote locations, scattered populations, and the high cost of building grid infrastructure. These regions often face physical and logistical barriers, including rugged terrain, long distances from existing power networks, and substantial investments required for construction and maintenance. By addressing these constraints, SE provides a practical and cost-effective solution, particularly through localized energy systems that meet the unique demands of such regions^3^. One of the most important applications of SE in rural settings is Photovoltaic Water Pumping Systems (PVWPS). These systems are used for irrigation, livestock watering, and other essential purposes, providing reliable and sustainable solutions that reduce dependency on traditional energy sources while enhancing agricultural productivity^4^.

PVWPS systems can be designed with or without batteries. While batteries store energy to ensure a reliable and constant power source for the pump, they have two significant drawbacks: technical issues and high costs^5^. To reduce the price of the studied PVWPS system, this paper suggests using a PVWPS system that operates without batteries. Instead, it incorporates a water storage tank, thereby avoiding the need for expensive batteries, typically the costliest component of a PV system^6^. The PVWPS typically comprises several key elements: a photovoltaic (PV) array (arranged in series and parallel), a power conditioning unit (which may include a DC/DC converter, a DC/AC inverter, or both), an electric motor, and a pump. The system operates by converting electrical energy produced by the PV array into mechanical energy through the electric motor. This mechanical energy is subsequently transformed into hydraulic energy by the pump. The selection of an appropriate electric motor to drive the pump is critical, as it directly influences the system’s overall performance. Motors are chosen based on several criteria: efficiency, availability, cost, and reliability. For instance, in^7^, the authors designed a PVWPS utilizing DC motors, which are appreciated for their simplicity in control and ability to connect directly to the PV system via a DC/DC converter. However, DC motors have notable drawbacks: their brushes and commutators are subject to wear, generate electrical noise, and necessitate regular maintenance. Moreover, DC motors typically operate at a fixed voltage, which limits their flexibility^8^. These limitations have prompted researchers to explore PVWPS configurations with AC motors, which offer higher efficiency and more excellent durability than DC motors. Among AC motors, Induction Motors (IM) are particularly recommended due to their robust and straightforward design, ease of maintenance, and repairability^9^. However, the nonlinear model of IMs complicates the control of its performances (speed, stator flux, and torque), which has prompted researchers to develop new control strategies to regulate IM behaviors^10^. The Scalar Control (SC) technique is one of the earliest control methods introduced in the literature, valued for its simplicity and cost-effectiveness^11^. However, SC does not directly regulate flux and torque, which reduces its efficiency and accuracy compared to more advanced methods. Field-oriented control (FOC) addresses these limitations by decoupling the motor’s flux and torque, ensuring excellent speed precision and rapid torque response across the entire speed range^12^. Nevertheless, system performance can be influenced by external load disturbances, uncertainties, and parameter fluctuations, mainly due to the reliance on multiple PI controllers. While robust in linear systems, these controllers are less effective in nonlinear systems^13^. In^14^, the authors developed a Sliding Mode Control (SMC) system that delivers high-precision control, making it suitable for precise control applications. However, SMC is prone to a phenomenon known as chattering, characterized by rapid oscillations in the control signals, which can cause vibrations and noise in the system. To overcome these challenges, researchers have explored Direct Torque Control (DTC), a technique introduced by Takahashi, Noguchi, and Depenbrock^15^.

DTC offers superior performance due to its simplicity, robustness under varying induction motor (IM) parameters, and elimination of the need for a current regulation loop. However, using hysteresis controllers in DTC can lead to significant fluctuations in electromagnetic torque and stator flux, especially at low speeds. These fluctuations can cause mechanical vibrations and increased noise. To address these issues, many researchers have focused on improving conventional DTC. To reduce flux and torque ripples in DTC applied to induction motors, the authors in^16^proposed replacing two-level inverters with multilevel inverters. While multilevel inverters enhance performance, they also increase costs due to additional power device requirements. In^9^, Space Vector Modulation (SVM) was proposed as an alternative. SVM enhances DTC by enabling precise control of the voltage and frequency applied to the motor, improving dynamic performance and efficiency. However, the accuracy of the PI controller and system parameters is critical to the success of SVM-DTC. Any inaccuracies can adversely affect performance and stability.

To further improve DTC, many researchers have integrated Artificial Intelligence (AI) techniques such as Artificial Neural Networks (ANNs)^17^, Fuzzy Logic (FL)^18^, and Genetic Algorithms (GAs)^19^. Recently, most studies have focused on DTC methods based on ANN. In^20^, the authors proposed an ANN algorithm to enhance Direct Torque Control (DTC) for Permanent Magnet Synchronous Motors (PMSMs). Simulation and real-time experiments conducted in MATLAB/Simulink and Dspace-1104 environments demonstrated the superior performance of ANN-DTC compared to conventional DTC, particularly in reducing torque and flux ripples. In^21^, an improved DTC method utilizing ANN with seven hysteresis controllers was introduced. The performance of the proposed method was evaluated by comparing simulation results with the conventional DTC approach, revealing the superiority and resilience of the new approach.

The power produced by the photovoltaic array is constantly affected by nonlinear characteristics (I-V) and (P-V), which vary with weather conditions such as irradiance, temperature, and shading^22^. To address these problems, Maximum PowerPoint Tracking (MPPT) techniques are implemented. These techniques can adjust the operating point to achieve maximum power, regardless of weather conditions^23^. Therefore, numerous MPPT techniques have been studied, which differ in efficiency, tracking speed, steady-state oscillations, difficulty, hardware implementation, global MPP tracking, and cost. MPPT is classified into two categories. The first category includes conventional methods such as Perturb and Observe (P&O)^24^ and Incremental Conductance (INC)^25^. Their simplicity and ease of implementation distinguish these methods. However, the problem with these methods is the continuous oscillations around the MPP, which cause a significant loss of power during the steady state^26^. The second category involves more recent techniques that are based on artificial intelligence (AI) methods, such as Fuzzy Logic Controllers (FLC)^27^, Artificial Neural Networks (ANNs)^28^, and Particle Swarm Optimization (PSO)^29^. Regardless of their complex structures, these methods perform better than conventional methods and can track the MPP under various conditions, especially partial shading and rapid environmental changes. ANN and FLC are the most commonly used AI methods based on MPPT^30^. These techniques offer advantages such as the ability to handle variations in input data, self-learning capabilities, and an adaptive nature suited for the nonlinear behavior of systems^31^. FLC-based MPPT trackers typically employ two inputs, the error and the change of error, along with five to seven fuzzy sets. However, FLC has limitations, including the time to reach the peak power point, the need for expert knowledge to design membership functions and control rules, and the inability to track the global MPP in partial shading conditions^32^. Using ANN algorithms has offered several key advantages that make them highly effective in maximizing the efficiency of PV systems, especially under challenging conditions like partial shading and rapidly changing environmental factors. These advantages include offline training, nonlinear mapping capabilities, high-speed response, robust operation, and reduced computational effort. Consequently, various ANN-based MPPT approaches have developed, each differing in controller configuration, required measurement signals, training algorithms, implementation complexity, and robustness^33^. In^34^ the authors introduced the ANN-based MPPT approach to optimize power output in photovoltaic systems, particularly electric vehicles, by only measuring voltages and currents. This method is cost-effective, requires no additional sensors, and adapts swiftly to fluctuating conditions. Simulations reveal its effectiveness and the trade-offs between measurement frequency and prediction accuracy. In^35^, the authors utilized experimental measurements of solar irradiance (Ir), temperature (T), and battery voltage (VBAT) as input data for the neural network, with the desired maximum power point (DMPP) serving as the output for a PV system equipped with a conventional MPPT device and a battery as a fixed load. Simulations confirm that this approach outperforms classical methods, making it a viable alternative for improving PV system efficiency. In^36^, the authors evaluated the performance of six ANN algorithms for MPPT in solar PV systems: Levenberg-Marquardt (LM), Broyden-Fletcher-Goldfarb-Shanno (BFGS), Resilient Propagation (RP), Gradient Descent with Momentum (GDM), and Scaled Conjugate Gradient (SCG). The study finds that LM and BFGS outperform the others in terms of convergence speed and overall performance, with LM identified as the leading algorithm for MPPT in solar PV systems.

Numerous configurations for PVWPS controllers have been proposed in the literature^37^. In^38^, the proposed PVWPS control system consists of a Fixed Step Size-P&O (FSS-P&O) for MPPT and SC for the IM. While FSS-P&O is valued for its simplicity and low cost, it encounters performance limitations due to its fixed step sizes. A small step size results in a slow response with fewer oscillations. In contrast, a significant step size provides a faster response with increased oscillation around the MPPT. The SC is simple and easy to implement. However, it has weak dynamic performance, particularly at low speeds. In^39^, the authors proposed Indirect Field-Oriented Control (IFOC) for IM and Variable Step Size-P&O (VSS-P&O) for MPPT. The IFOC depends on mechanical speed and requires accurate knowledge of motor parameters, which adds to its complexity. While VSS-P&O results in less oscillation. However, tracking remains an issue at the initial step change. In^40^, DTC proposed to control the IM instead of IFOC and VSS-P&O for MPPT. Despite its simplicity and robustness, DTC has disadvantages, such as torque and flux ripples. In^41^, the authors proposed the implementation of Variable Step Size-INC (VSS-INC) for PV array operation and 12-sector DTC for induction motor control. VSS-INC outperforms VSS-P&O in terms of performance. However, this method can only track local MPP instead of global MPP, which may take a long time and have low tracking accuracy. The 12-sector DTC method has advantages over the 6-sector DTC method, such as better torque control and fewer torque ripples. However, working with 12-sector DTC involves higher computational requirements, increased switching frequency, and higher harmonic distortion. These conventional methods applied to the MPPT controller and the induction motor controller in PVWPS have shown significant potential; however, they also possess inherent limitations. To address the challenges in PVWPS, this paper introduces advanced strategies based on Artificial Neural Networks. ANNs are highly adaptive and flexible tools capable of learning from complex datasets and responding dynamically to changing conditions. Their ability to handle non-linear behaviors and real-time variations makes them an effective solution for improving system performance. By leveraging ANNs, the system’s efficiency, accuracy, and stability can be significantly enhanced. This paper proposes the application of ANNs to optimize two critical controllers of the PVWPS: the Maximum Power Point Tracking (MPPT) controller and the Direct Torque Control (DTC) controller for the induction motor.

- ANN-Based MPPT Controller: The proposed ANN-MPPT controller is designed to maximize the power output of the PV array, even under challenging conditions such as rapid changes in solar irradiance, temperature, and partial shading. Unlike conventional MPPT techniques, which may exhibit slower responses and power oscillations, the ANN-MPPT algorithm uses real-time data to learn and adapt dynamically. This approach enables faster and more precise tracking of the maximum power point (MPP), thereby improving the overall efficiency of the PVWPS.
- ANN-Enhanced DTC Controller: The ANN is also integrated into the DTC controller to address the limitations of conventional DTC, such as torque and flux ripples and high distortion of stator currents. In this approach, the ANN replaces key components of the traditional DTC system, including the flux and torque comparators, PI speed controller, and switching tables. By optimizing the control strategy, the ANN-based DTC improves the performance of the induction motor, ensuring smoother torque and flux control, better speed precision, and reduced electromagnetic disturbances.
- Combined Impact of ANN-MPPT and ANN-DTC: By integrating ANN-based strategies into the MPPT and DTC controllers, the PVWPS benefits from comprehensive improvements in performance. The ANN-MPPT controller increases the PV array’s energy efficiency, while the ANN-DTC minimizes mechanical and electrical losses in the motor. These advancements enhance the system’s stability, reliability, and water-pumping capability.

The paper is structured as follows: Section “Solar water pumping system design” provides a comprehensive overview of the Photovoltaic Water Pumping System and its key components. Section “Strategies for the control of the system” delves into various control strategies, focusing on the ANN-MPPT and ANN-DTC. Section “Simulation results and interpretatio” outlines the simulation process and offers a detailed analysis of the results. Section “Real-time simulation and interpreting” presents the Real-Time simulation and visualization and a discussion. Section “Conclusion” concludes the study and suggests future research directions.

## Solar water pumping system design

**Fig. 1:**
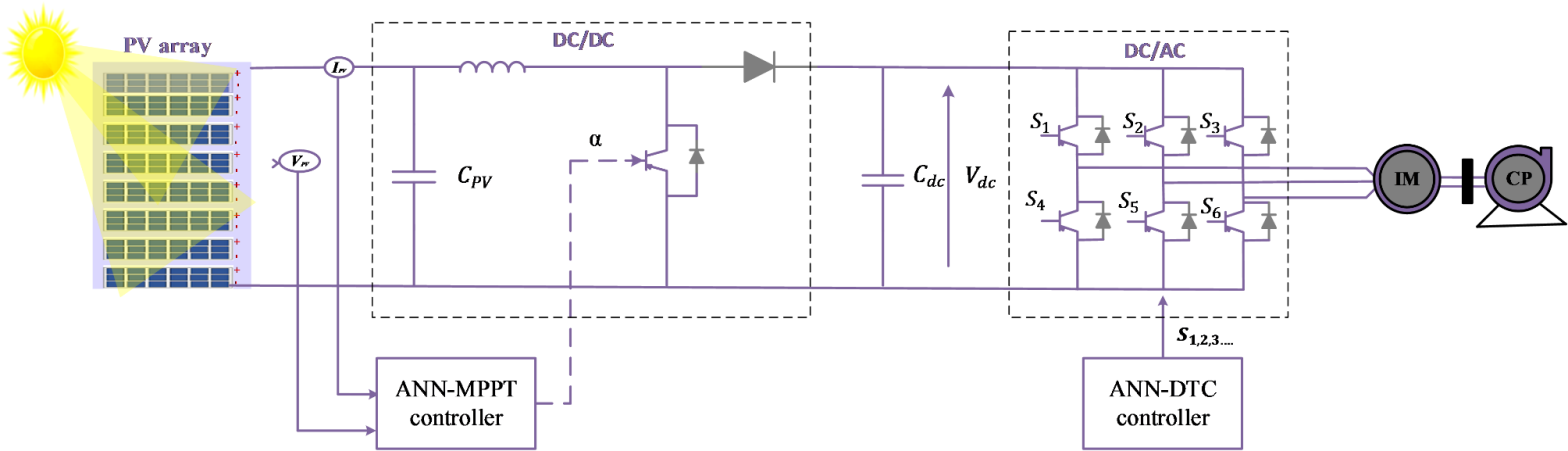
Schematic diagram of Photovoltaic Water Pumping Systems.

Figure 1 presents the overall schematic of the studied system. It consists of a 1.8 kW PV array made up of 8 series-connected SunPower T5-SER-235P modules with the characteristics displayed in Table A.1, a DC-DC boost converter, a VSI, and a 1.5 kW three-phase induction motor connected to a centrifugal pump. An ANN-based MPPT is used to maximize power extraction from the PV array. Meanwhile, the DTC and ANN-DTC are used to adjust the induction motor.

### Architecture of the PV panel

**Fig. 2:**
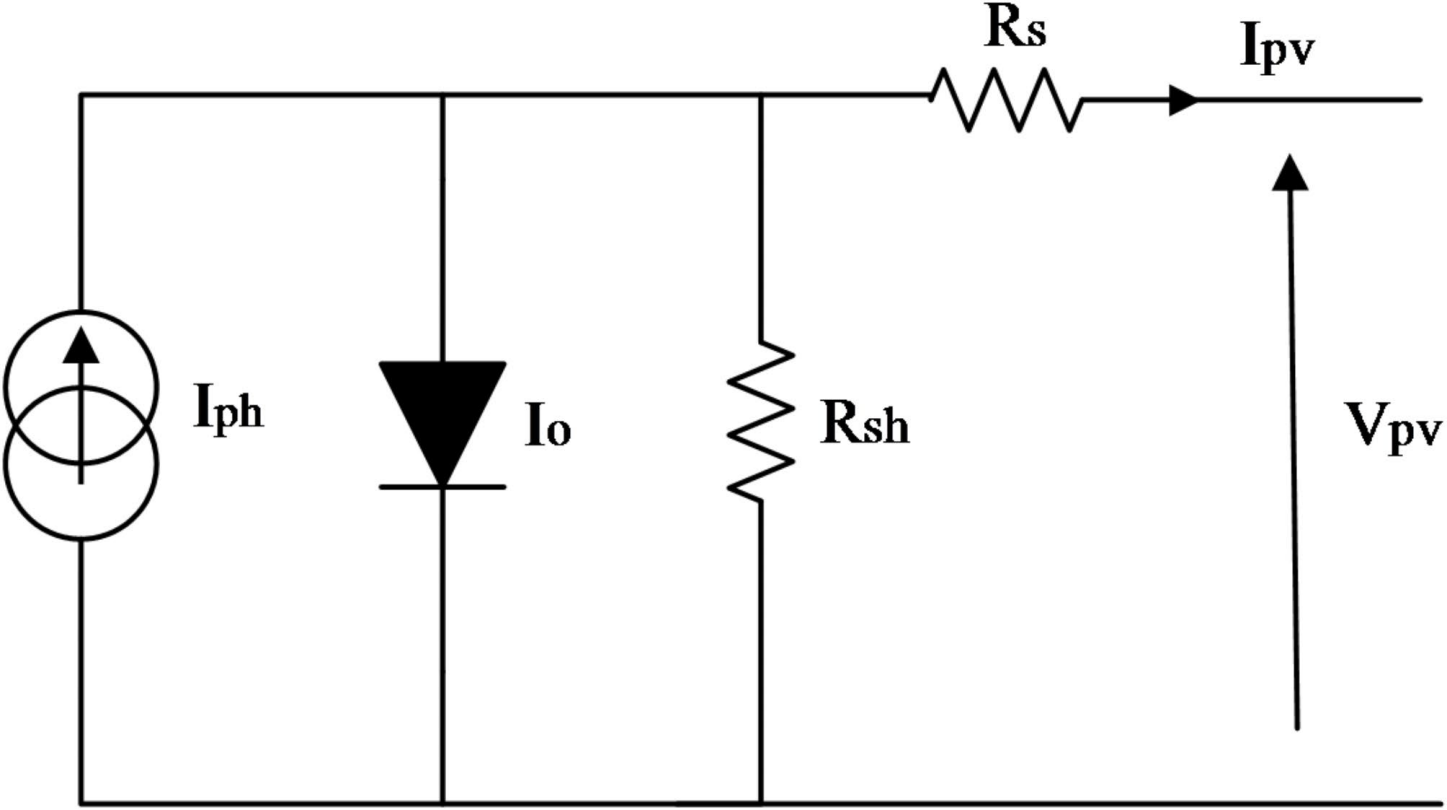
The equivalent circuit model of the PV panel.

As depicted in Fig. 2, the equivalent circuit of the PV panel model includes a current source representing the photon current, which operates in parallel with a diode (D) and a shunt resistor ($\:{R}_{sh}$). All of these components are connected to a series resistor ($\:{R}_{s}$)^1^.

The current (I) and voltage (V) mathematical expressions generated by the PV panel are given below:

1 $$\:{I}_{pv}={I}_{ph}-{I}_{s}\left({e}^{\frac{q\left({V}_{pv}+{R}_{s}{I}_{pv}\right)}{aKT{N}_{s}}}-1\right)-\frac{({V}_{pv}+{I}_{pv}{R}_{s})}{{R}_{sh}}$$

2 $$\:{I}_{ph}=({I}_{sc}+{K}_{i}\left(T-298.15\right)\frac{G}{1000}$$

3 $$\:{I}_{s}=\frac{{I}_{sc}+{K}_{i}(T-298.15)}{{e}^{\frac{q({V}_{oc}+{K}_{v}(T-298.15)}{aKT{N}_{s}}}-1}$$

### Design of DC-DC boost converter

**Fig. 3:**
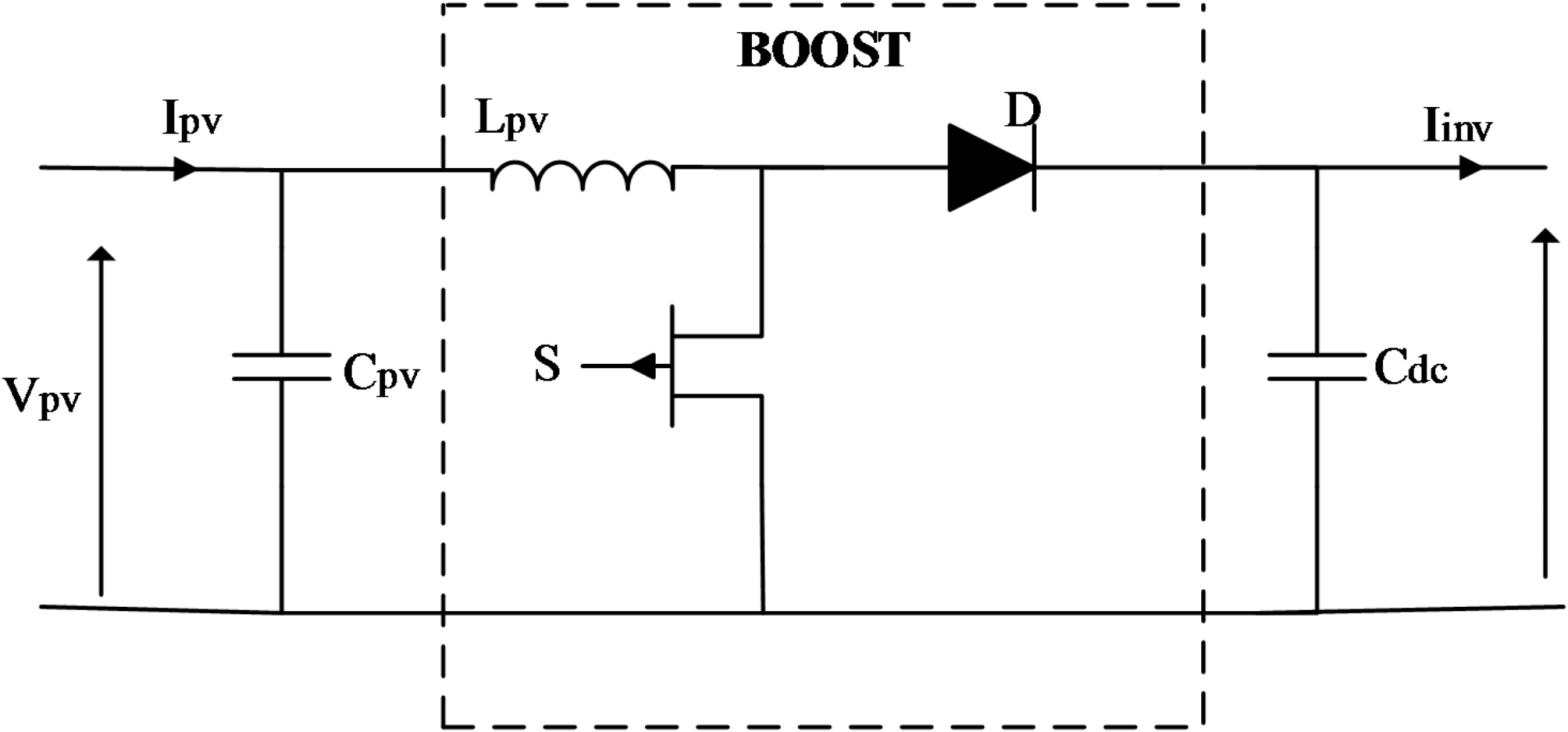
Design of the DC/DC boost converter.

The corresponding circuit of the DC/DC boost converter is depicted in Fig. 3, which is positioned between the photovoltaic arrays and the Voltage Source Inverter to amplify the voltage and optimize the panels’ power output. the formulas used to determine the parameters of the boost converter are summarized in Table A.2.

### Voltage source inverter

**Fig. 4:**
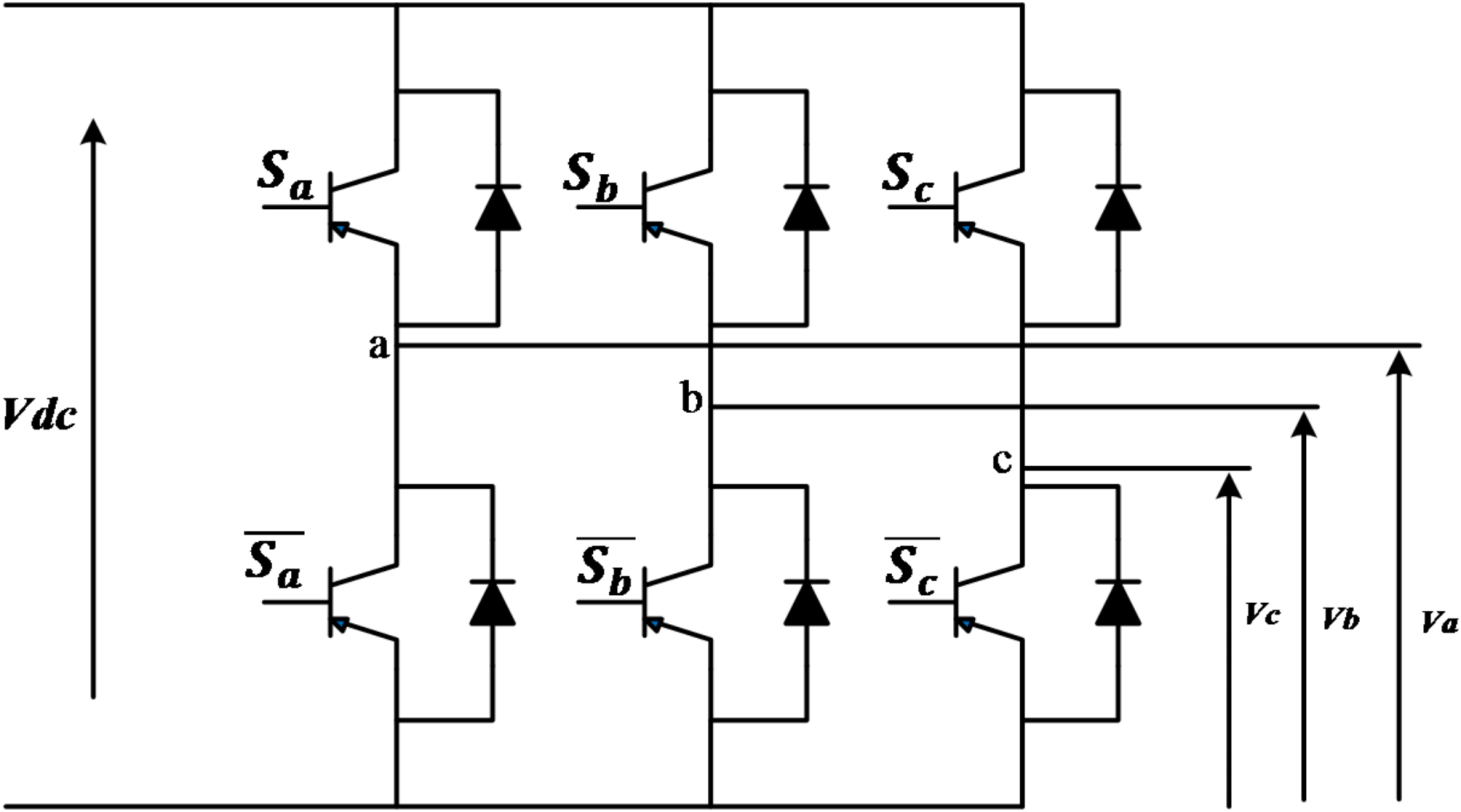
Equivalent Circuit of the VSI.

The VSI is a type of regulator that converts DC voltage into an adjustable frequency and magnitude of AC voltage. As shown in Fig. 4, the VSI comprises six IGBT switches regulated by analog values^4^.

The inverter’s output voltages can be expressed as follows:

4 $$\:\left[\begin{array}{c}{V}_{a}\\\:{V}_{b}\\\:{V}_{c}\end{array}\right]=\frac{{V}_{dc}}{2}\:\left[\begin{array}{ccc}2&\:-1&\:-1\\\:-1&\:2&\:-1\\\:-1&\:-1&\:2\end{array}\right]\left[\begin{array}{c}{S}_{a}\\\:{S}_{b}\\\:{S}_{c}\end{array}\right]$$

### Induction motor mathematic model

**Fig. 5:**
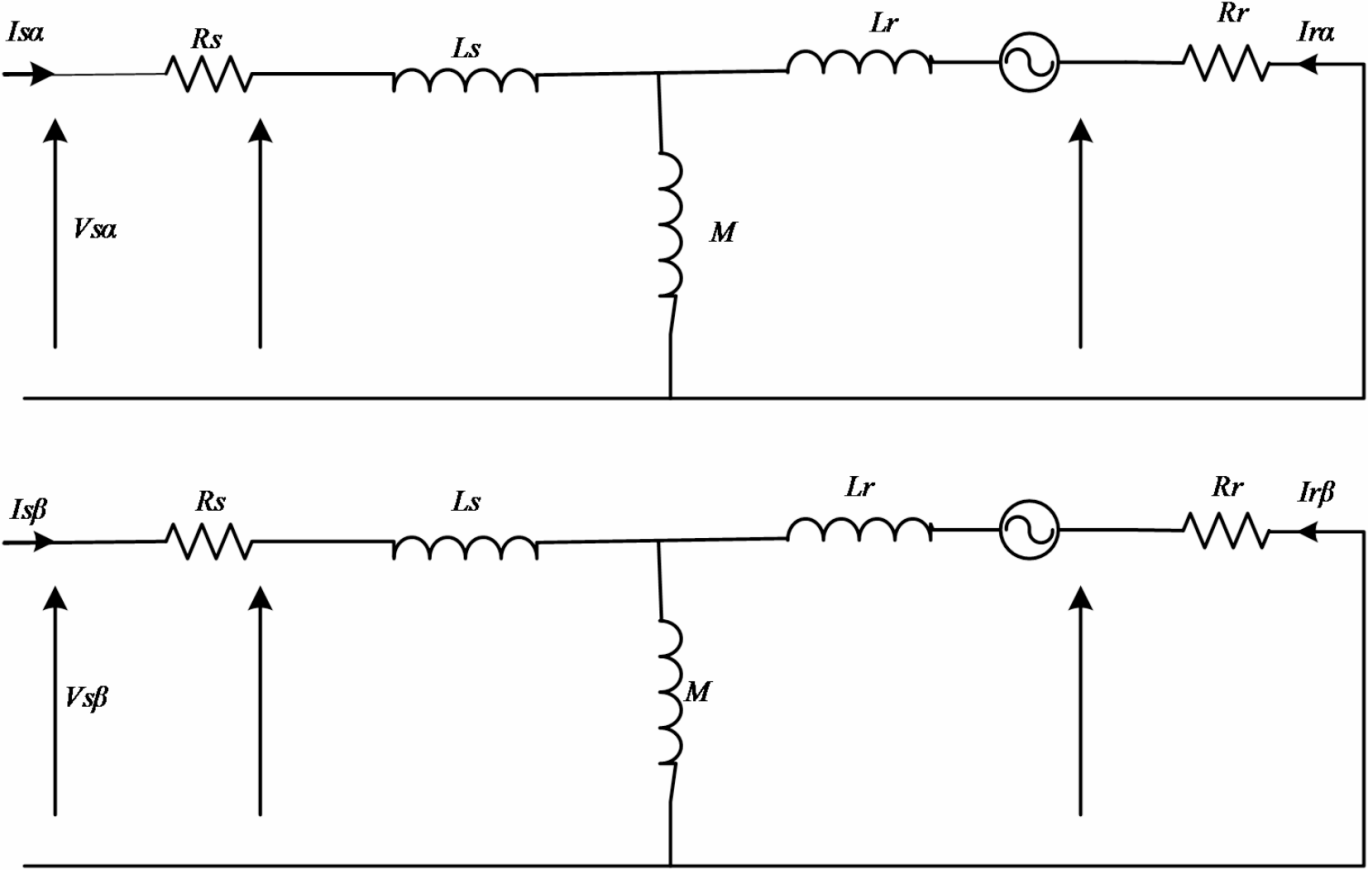
Electrical circuit diagram of the IM in the (α, β) reference frame.

The mathematical equations employed for describing the IM in the (α, β) frame are presented in Fig. 5 and can be expressed as follows^16^:

- The stator voltage in the (α, β) frame:
  5 $$\:\left\{\begin{array}{c}{V}_{s\alpha\:}={R}_{s}{I}_{s\alpha\:}+\frac{d{{\varnothing}}_{s\alpha\:}}{dt}\\\:{V}_{s\beta\:}={R}_{s}{I}_{s\beta\:}+\frac{d{{\varnothing}}_{s\beta\:}}{dt}\:\:\end{array}\right.$$
- The rotor voltage in the (α, β) frame:
  6 $$\:\left\{\begin{array}{c}0={R}_{r}{I}_{r\alpha\:}+\frac{d{{\varnothing}}_{r\alpha\:}}{dt}+{\omega\:}_{m}{{\varnothing}}_{r\beta\:}\:\:\:\:\:\:\\\:0={R}_{r}{I}_{r\beta\:}+\frac{d{{\varnothing}}_{r\beta\:}}{dt}+{\omega\:}_{m}{{\varnothing}}_{r\alpha\:}\:\:\:\:\:\:\end{array}\right.$$
- The stator flux in the (α, β) frame:
  7 $$\:\left\{\begin{array}{c}{\:\:{\varnothing}}_{s\alpha\:}={L}_{s}{I}_{s\alpha\:}+M{I}_{r\alpha\:}\:\:\:\\\:{{\varnothing}}_{s\beta\:}={L}_{s}{I}_{s\beta\:}+M{I}_{r\beta\:}\end{array}\right.$$
- The rotor flux in the (α, β) frame:
  8 $$\:\left\{\begin{array}{c}{{\varnothing}}_{r\alpha\:}={L}_{r}{I}_{r\alpha\:}+M{I}_{s\alpha\:}\:\:\:\\\:{{\varnothing}}_{r\beta\:}={L}_{r}{I}_{r\beta\:}+M{I}_{s\beta\:}\:\:\:\:\:\:\end{array}\right.$$
- The electromagnetic torque and its movement in the frame (α, β) are expressed by the following equations:
  9 $$\:\left\{\begin{array}{c}{T}_{e}=\frac{3}{2}\times\:p\left({I}_{s\beta\:}{\varnothing\:}_{s\alpha\:}-{I}_{s\alpha\:}{\varnothing\:}_{s\beta\:}\right)\:\\\:{T}_{e}-{T}_{r}=J\frac{d\varOmega\:}{dt}+f\varOmega\:\end{array}\right.$$

### Centrifugal pump

The chosen centrifugal pump load torque $\:{T}_{p}$is considered to be proportionate to the square of the IM rotor speed (Ω), as given in Eq. (10) ^42^.

10 $$\:{T}_{p}=\:{K}_{pump}{\varOmega\:}^{2}$$

where $\:{K}_{pump}$ is the proportionality constant of the pump and is calculated as:

11 $$\:{K}_{pump}=\frac{{T}_{P}}{{\varOmega\:}^{2}}=\frac{10}{{\left(148\right)}^{2}}=0.00045653\text{N}\text{m}/{(\text{r}\text{a}\text{d}/\text{s})}^{2}$$

By applying the similarity laws^21^, centrifugal pumps can establish the characteristics of centrifugal pumps (P, H, Q) for a rotation speed N to derive the novel characteristic ($$\:{P}_{n}$$$$\:{H}_{n},$$ $$\:{Q}_{n})$$ for any rotation speed using the following equations:

12 $$\:{Q}_{n}=Q\times\:\:\left(\frac{{N}_{n}}{N}\right)$$

13 $$\:{H}_{n}=H\times\:\:{\left(\frac{{N}_{n}}{N}\right)}^{2}$$

14 $$\:{P}_{n}=P\times\:{\left(\frac{{N}_{n}}{N}\right)}^{3}$$

## Strategies for the control of the system

**Fig. 6:**
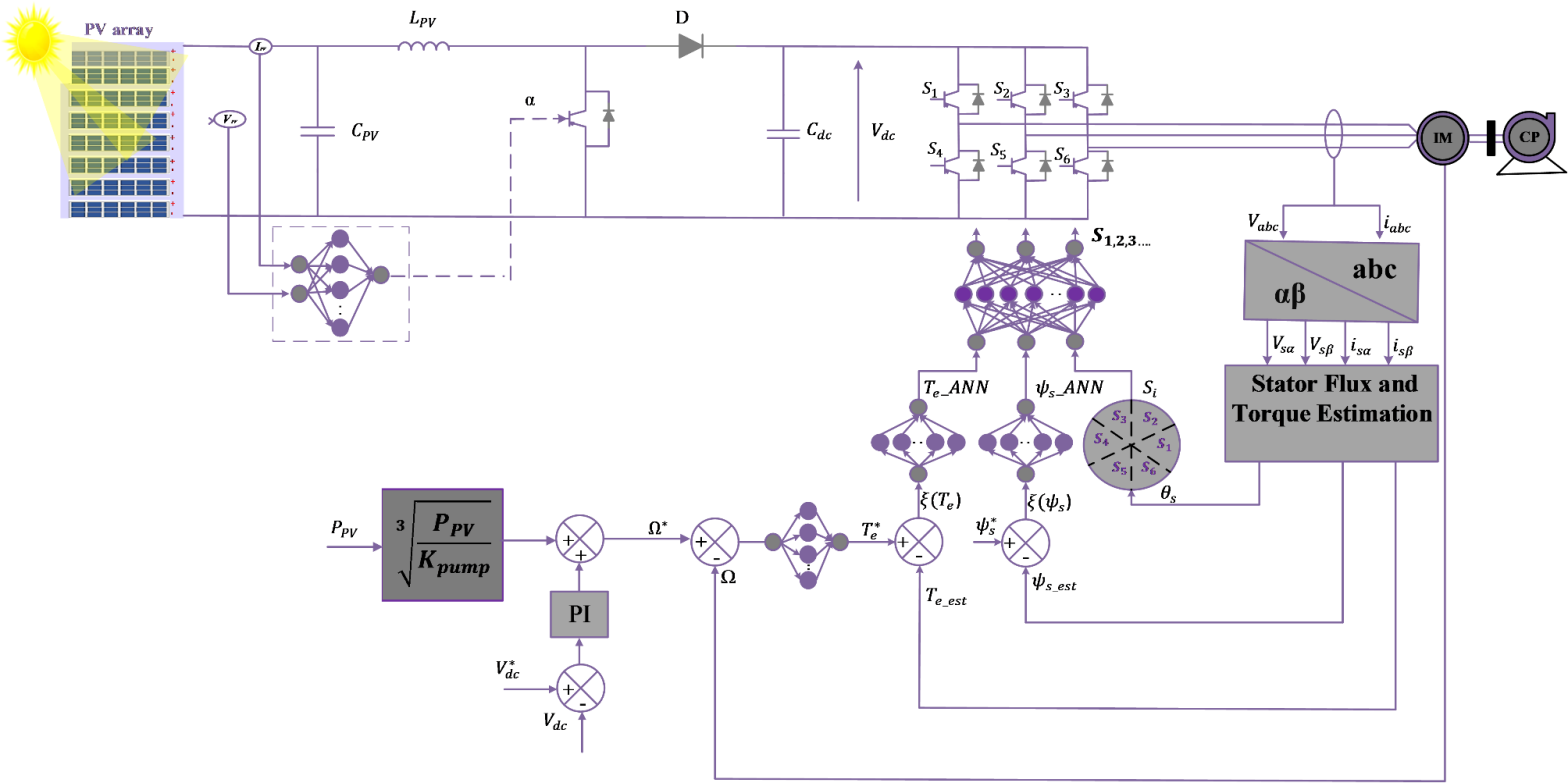
Presented configuration for the photovoltaic water pumping systems.

To explain the functioning of the suggested control for the PVWPS depicted in Fig. 6. This section of the paper is structured into three parts. The first part outlines the general principles behind ANNs, the second part introduces MPPT controllers based on ANN, and the last part studies DTC based on ANN.

### Principle of the artificial neural network

Artificial neural networks are computational models inspired by the structure and function of the neural networks in the human brain. These networks rely on training data sets to identify patterns in the input data, which are then tested or evaluated during the validation phase. ANNs are distinguished by their capability to learn complex, non-linear input/output relationships, rendering them highly effective in addressing non-linear problems across various domains^5^. Moreover, ANNs offer inherent advantages such as high noise immunity and robustness, making them less vulnerable to changes in operating conditions compared to conventional engineering approaches^6^.

#### Artificial neural

**Fig. 7:**
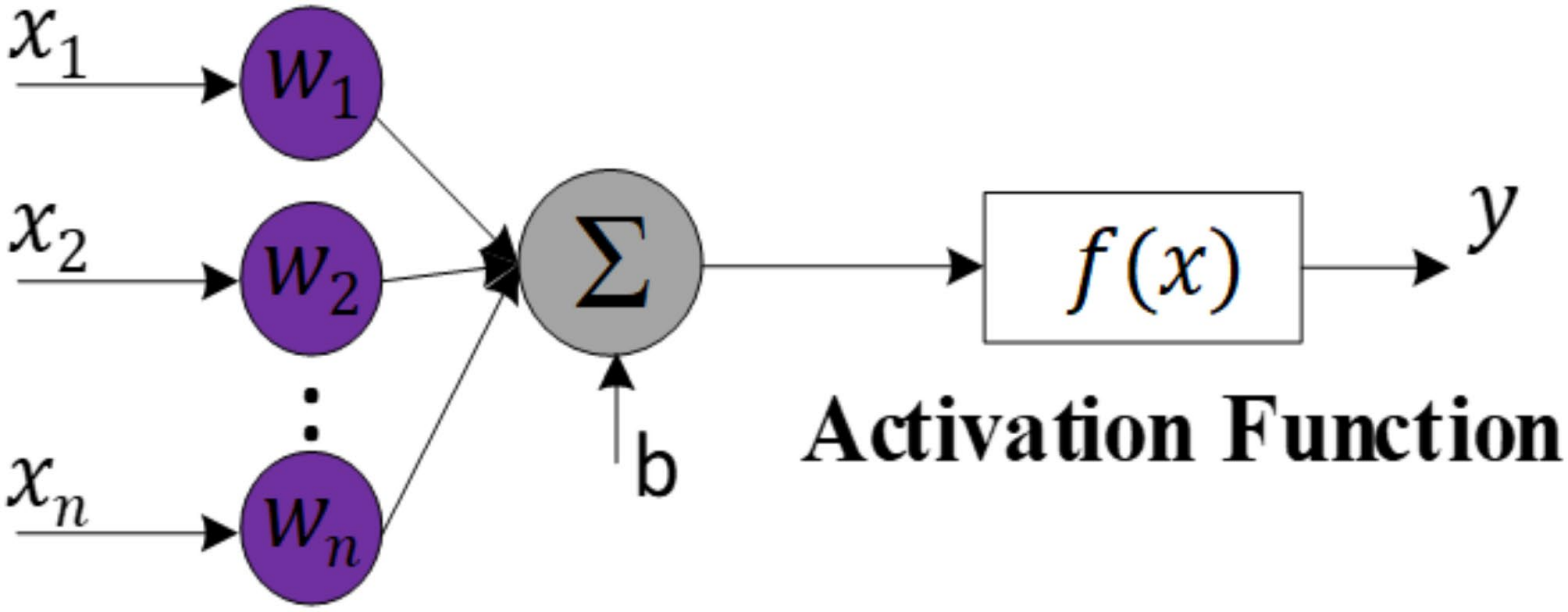
Artificial Neural Architecture.

The fundamental components of the ANN are the neurons. Each neuron integrates information from other neurons through multiple inputs, with each input having an associated adjustable weight. This integration process is achieved through a summation function (Σ). The resulting sum is then passed through an activation function to determine the neuron’s output, as seen in Fig. 7. The neuron’s behavior can be described mathematically by the following equation:

15 $$\:\text{y}=f\left(\sum\:_{i=1}^{n}{w}_{i}{x}_{i}+\text{b}\right)$$

where $\:x$ and $\:y$ represent the input and output of the neuron, respectively, $\:{w}_{i}$ represents the weight coefficients of the connections between the inputs and neuron i, and b is the bias of neuron i. $\:f$ is the activation function, which plays a crucial role in neural networks. Common choices for activation functions include sigmoid (also called logistic function), tanh (hyperbolic tangent), and rectified linear unit (ReLU).

#### Artificial neural network architecture

**Fig. 8:**
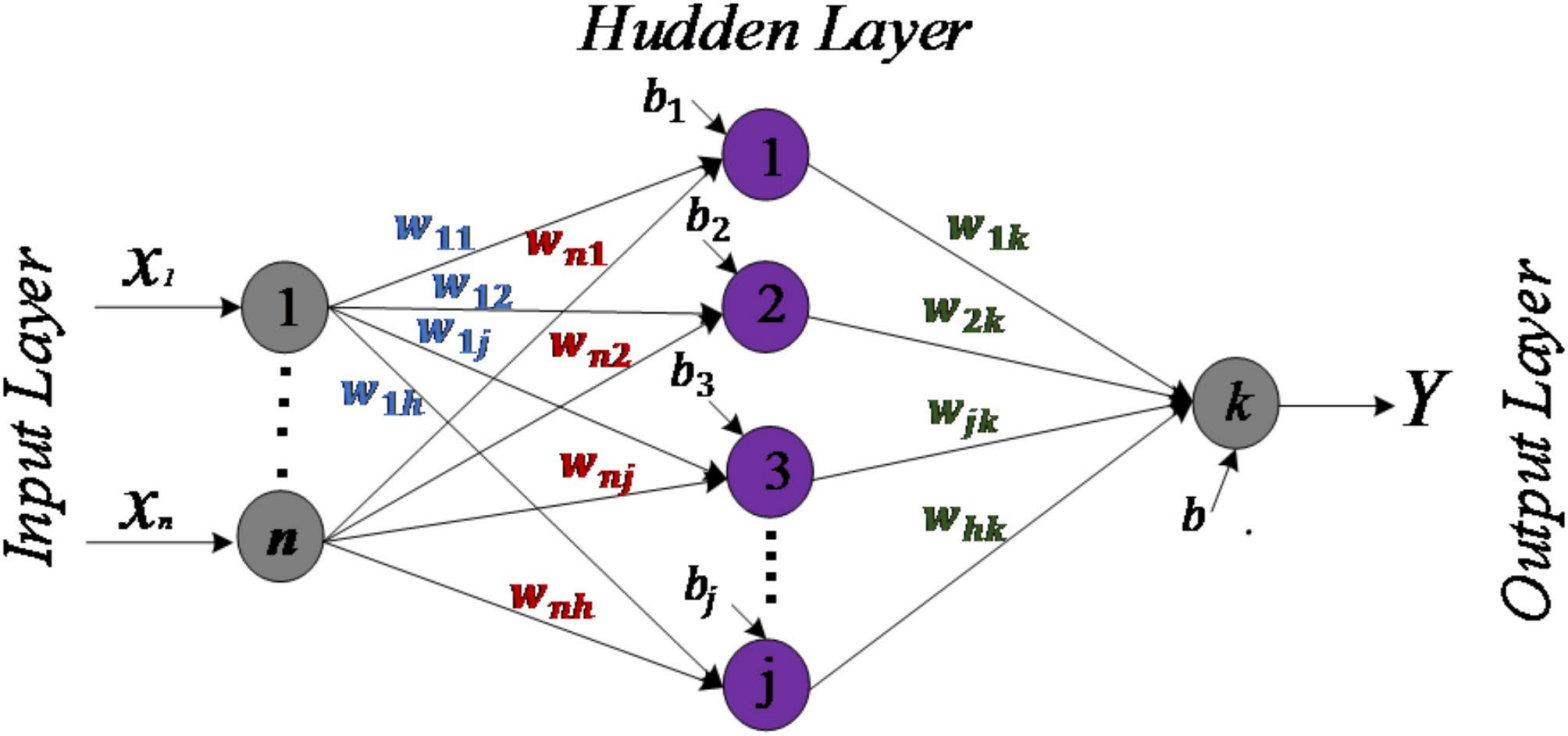
Standard structure of the artificial neural network.

Several neural network architectures exist, including simple perceptrons, multilayer perceptrons (MLPs), the ADALINE model, Hopfield networks, and Kohonen networks^23^. Among these, MLPs (see Fig. 8) are particularly noteworthy for overcoming the limitations of single-layer perceptrons, such as their inability to solve non-linear problems. MLPs achieve this by introducing one or more hidden layers between the input and output layers, allowing them to handle more complex tasks. From Fig. 8, the outputs of the hidden and output layers can be determined using the following mathematical equations:

- Hidden layer outputs:
  16 $$\:{H}_{j}=f\left({v}_{j}\right)=\frac{1}{1+{\text{e}}^{{\text{v}}_{\text{j}}}}$$
  where
  17 $$\:{v}_{j}={\text{b}}_{\text{j}}+\sum\:_{\text{i}=1}^{\text{n}}{\text{x}}_{\text{i}}\text{*}{\text{w}}_{\text{i}\text{j}}$$
- Output Layer Output:
  18 $$\:{Y=b}_{k}+\sum\:_{j=1}^{h}{H}_{j}*{w}_{jk}$$
  $\:f$ is a nonlinear activation function for neuron j in the hidden layer, which can be either a sigmoid function (as shown in Eq. 16) or a hyperbolic tangent (tanh) function. Meanwhile, the output y includes a linear activation function for the output neuron.

The neural network’s training data constitute one of the main features of ANN for learning and enhancing its performance, which uses training functions such as gradient descent backpropagation, one-step secant backpropagation, BFGS quasi-Newton backpropagation, and Levenberg‒Marquardt backpropagation to adjust and update the weights of the layers with the minimum Mean Squared Error (MSE) which is expressed by Eq. (19)^24^.

19 $$\:MSE=\frac{1}{M}\sum\:_{k=1}^{M}{\left({Y}_{i}-{\widehat{Y}}_{i}\right)}^{2}$$

where$\:\:\:{Y}_{i}$ represents the actual target output, $\:{\widehat{Y}}_{i}$ is the predicted output, and N is the total number of input-output training samples.

Equation (20) presents the expression used to update the weights of each neuron during the training process. This process is crucial for improving neural network performance by gradually adjusting the weights to decrease the cost function value (MSE).

20 $$\:{\omega\:}_{n}\left(k+1\right)={\omega\:}_{n}\left(k\right)-\eta\:\frac{\partial\:MSE\left(k\right)}{\partial\:{\omega\:}_{n}\left(k\right)}$$

### MPPT-based ANN

**Fig. 9:**
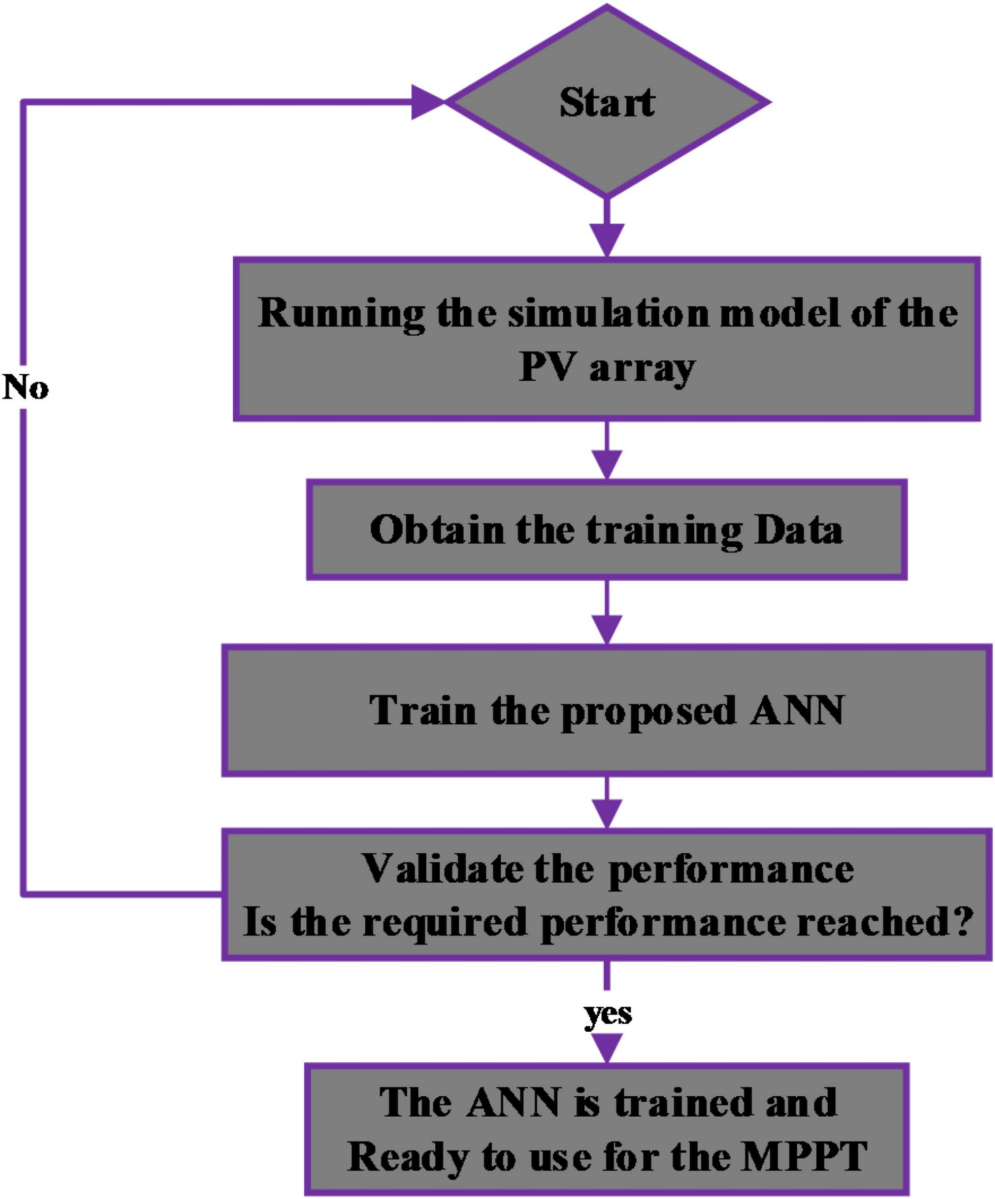
Flowchart of ANN For MPPT during the training process.

MPPT controller is a technique employed to improve the efficiency of the PVWPS. It allows PV panels to generate the maximum amount of power possible, regardless of environmental factors including irradiation, temperature, and shading. Therefore, numerous MPPT methods have been recently suggested, each with its own specifications, limitations, and applications^22^. Among these methods, the ANN-based MPPT controller has garnered significant attention from researchers due to its exceptional performance in identifying and estimating unknown parameters. The input layers of ANN-based MPPT controller can include PV panel characteristics such as $\:{V}_{PV}$ and $\:{I}_{PV}$, or the environmental data such as irradiation and temperature, or any combination of these parameters. The output layer usually includes the duty cycle. The accuracy and speed of the ANN-based MPPT can be optimized by using an appropriate method for the hidden layer and ensuring proper training of the ANN. The flowchart of ANN-MPPT is presented in Fig. 9.

#### Data collection for the ANN-MPPT

**Fig. 10:**
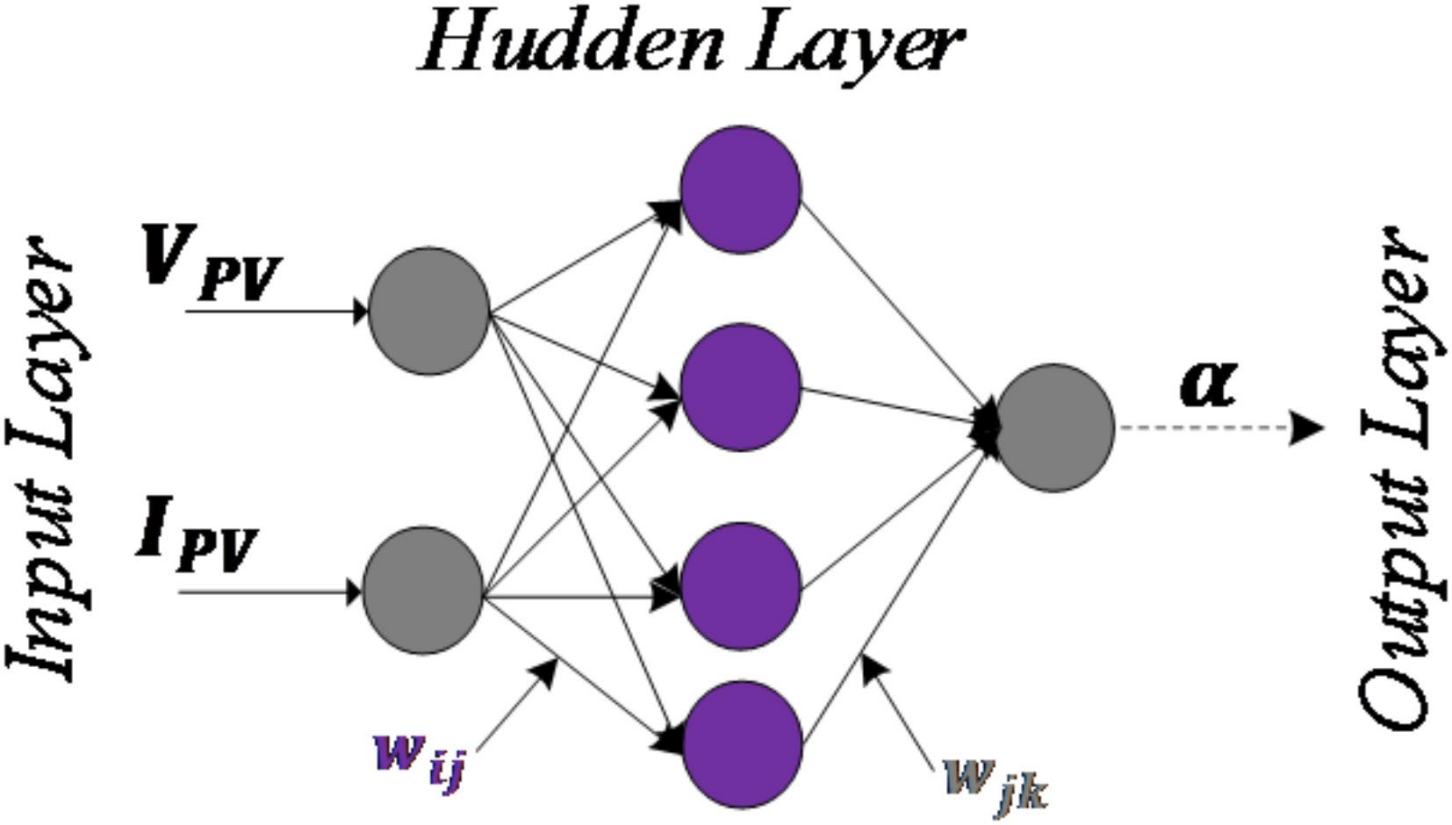
Proposed structure for the ANN-based MPPT algorithm.

The proposed structure of the ANN-based MPPT algorithm in this study is depicted in Fig. 10, which uses photovoltaic voltage ($\:{V}_{PV}$) and current ($\:{I}_{PV}$) as input variables to determine the duty cycle (α) for controlling the DC-DC converter. The neural network dynamically adjusts the duty cycle in response to fluctuations in $\:{V}_{PV}$ and $\:{I}_{PV}$caused by varying weather conditions, ensuring efficient and reliable tracking of the maximum power point (MPP). The training data for this ANN model were obtained by loading input-output sample data patterns generated by the workspace from the Incremental Conductance (INC) algorithm simulation outcomes on MATLAB/Simulink. These simulations produced a range of $\:{V}_{PV}$and $\:{I}_{PV}$ values under different solar irradiation levels and the corresponding optimal duty cycle values. This simulated dataset was then used to train the ANN, enabling it to learn the relationship between the input variables and the optimal duty cycle required to achieve MPP. Since there is no definitive rule for selecting the number of hidden layers and neurons in the neural network, a trial-and-error approach was employed. The process began with a simple structure (one hidden layer with a small number of neurons) and gradually increased the number of neurons while evaluating performance at each step. The hidden layer utilized tangent-sigmoid activation functions, while the output layer employed a linear activation function to provide the duty cycle value.

#### Network training for the ANN-MPPT

The network training was performed in the MATLAB nntraintool. Feed-forward networks with a back-propagation Levenberg‒Marquardt algorithm adjusted weights with a minimum error. The data were split into 70% for training, 15% for testing, and the rest for validation. The neural network parameters are presented in Table 1.

**Table 1:** Parameters of the network.

Type of network	Feed-forward
Activation function of Hidden layer	Sigmoid
Output layer activation function	Linear
Back-propagation algorithm	Levenberg‒Marquardt
Number of the input neurons	2
Number of hidden neurons	10
Number of output neurons	1
Number of epochs	1000
Learning rate	0.5

**Fig. 11:**
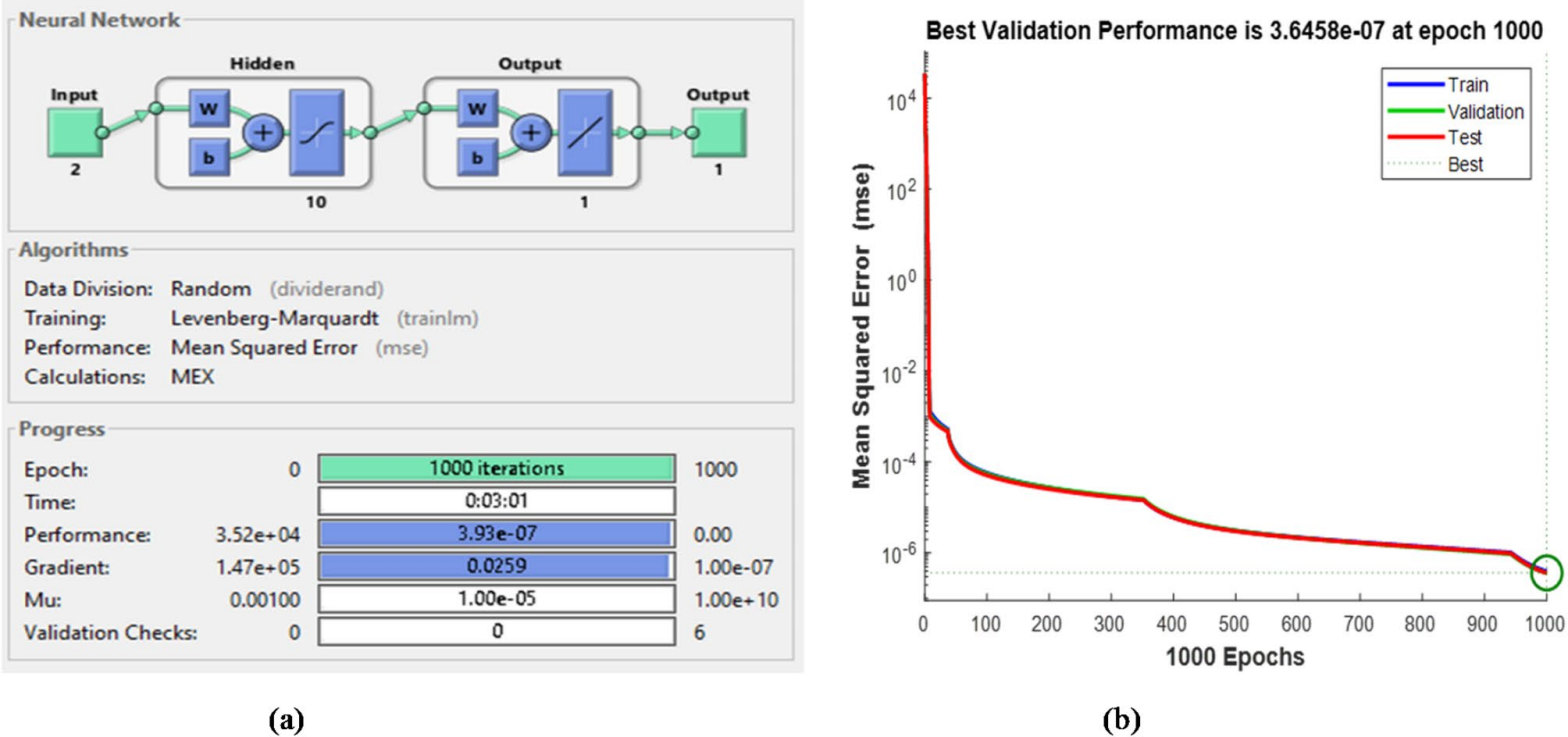
(**a**) ANN-based MPPT controller architecture and its training evolution (**b**) Mean square error performance.

Figure 11a,b show the training outcomes for the ANN-based MPPT controller block, where Fig. 11a represents the 2-10-1-1 architecture used, while Fig. 11b illustrates the development of the MSE (training, testing, validation), which decreases rapidly as the number of epochs increases until it reaches 3.64 × 10 − 7 at 1000 epochs. Figure 11a also demonstrates the convergent behavior of the training, validating, and testing samples, indicating successful training of the network and a close alignment between the neural network’s output and the desired output.

## The direct torque control concept of IM based on ANN

### Direct torque control

#### Principle of direct torque control

DTC is a control technique introduced by Takahashi in 1986 and is considered one of the high-performance strategies used for water pumping systems. This technique enables direct and independent regulation of the torque and flux of the induction motor by selecting the most appropriate voltage vector from a specified switching table. The selection process involves maintaining torque amplitudes and flux levels using two-level and three-level hysteresis comparators, these comparators receive inputs from flux and torque errors, which are determined by comparing the reference values with their estimated values. The outputs of these comparators, combined with information about the position of the flux vector, are used to identify the appropriate switching vector^26^.

#### Electromagnetic torque and stator flux estimation

##### Estimation of the stator flux

The estimation components of stator flux$\:\:{{\varnothing}}_{s\alpha\:}$ and $\:{{\varnothing}}_{s\beta\:}$ can be determined as follows:

21 $$\:{{\varnothing}}_{s\alpha\:}={\int\:}_{0}^{t}\left({V}_{s\alpha\:}-{R}_{s}{I}_{s\alpha\:}\right)dt$$

22 $$\:{{\varnothing}}_{s\beta\:}={\int\:}_{0}^{t}\left({V}_{s\beta\:}-{R}_{s}{I}_{s\beta\:}\right)dt$$

where the stator voltages in the (α, β) reference frame depend on the $\:{V}_{dc}$ voltage and the switch state ($\:{S}_{a},$ $\:{S}_{b},$ $\:{S}_{c}$) and are expressed as follows:

23 $$\:{V}_{s\alpha\:}=\sqrt{\frac{2}{3}}{V}_{dc}({S}_{a}-\frac{1}{2}\left({S}_{b}+{S}_{c})\right)$$

24 $$\:{V}_{s\beta\:}=\sqrt{\frac{1}{2}}{V}_{dc}({S}_{b}-{S}_{c})$$

##### Estimation of the torque

The estimated electromagnetic torque can be determined by using the estimated flux and currents as follows:

25 $$\:{T}_{e}=\frac{3}{2}\times\:p\left({I}_{s\beta\:}{{\varnothing}}_{s\alpha\:}-{I}_{s\alpha\:}{{\varnothing}}_{s\beta\:}\right)\:\:$$

where the stator currents ($\:{I}_{s\alpha\:}$,$\:{I}_{s\beta\:}$) are obtained by performing a Concordia transform to the measurement currents $\:{I}_{sa}$, $\:{I}_{sb}$ and $\:{I}_{sc}$ of the IM as follows:

26 $$\:{I}_{s\alpha\:}=\sqrt{\frac{2}{3}}{I}_{sa}$$

27 $$\:{I}_{s\beta\:}=\:\frac{1}{\sqrt{2}}({I}_{sb}-{I}_{sc})$$

#### Flux and electromagnetic torque hysteresis controller

**Fig. 12:**
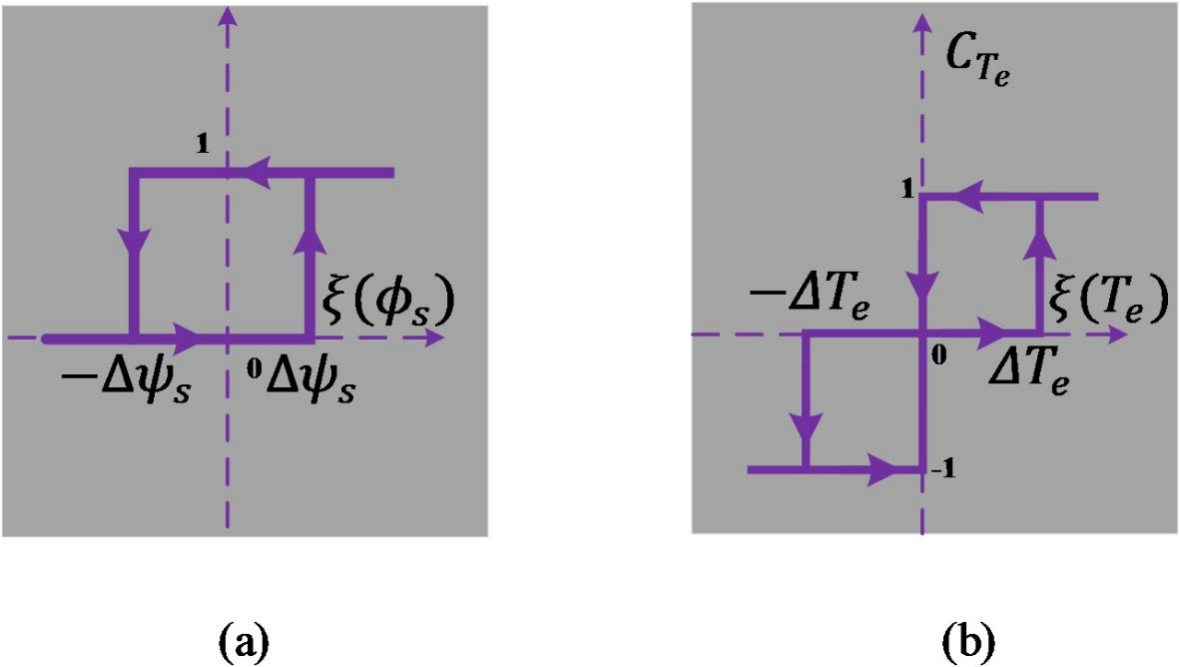
Hysteresis controllers for two-level stator flux (**a**) and three-level electromagnetic torque (**b**).

A two-level hysteresis comparator (see Fig. 12a) is used for flux regulation. This controller aims to maintain the magnitude of the stator flux vector ($\:{\varnothing\:}_{s}$) on a circular trajectory. Additionally, a three-level hysteresis comparator (Fig. 12b) is used for torque regulation to control the motor in both directions of rotation.

#### Description of the switching table and their voltage vectors

**Fig. 13:**
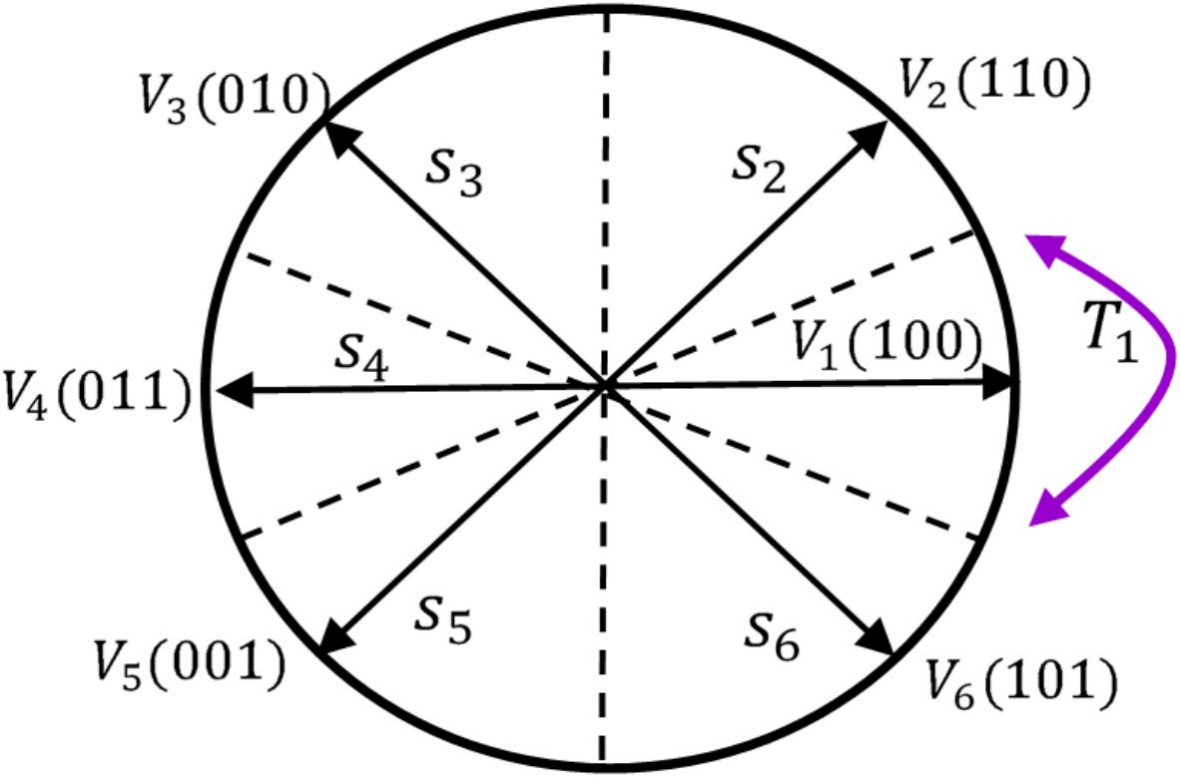
Controlling stator flux with an appropriate voltage vector Vi from (1 to7).

The switching table is a basic component of the DTC that selects the optimal voltage vector (Table 2) from eight voltage vectors, of which two are zeros (V_0_, V_7_) and six are activated (V_1_ to V_6_). These voltages are split into six sectors (S_1_, S_2_, S_3_, S_4_, S_5_, S_6_), as shown in Fig. 13. The selection of the optimal voltage vector relies on the input of the switching table, which is the stator flux position, and the output of the flux and torque hysteresis controllers.

**Table 2:** The conventional DTC switching table.

Flux$\:{\:\:\varDelta\:{\varnothing}}_{e}$	1			0		
Torque$\:\:{\varDelta\:T}_{e}$	1	0	-1	1	0	-1
Sectors (Si)						
1	$\:{V}_{2}$	$\:{V}_{0}$	$\:\:\:{V}_{6}$	$\:{\:\:\:\:V}_{3}$	$\:{V}_{7}$	$\:{V}_{5}$
2	$\:{V}_{3}$	$\:{V}_{7}$	$\:{\:V}_{1}$	$\:{\:\:\:\:V}_{4}$	$\:{V}_{0}$	$\:{V}_{6}$
3	$\:{V}_{4}$	$\:{V}_{0}$	$\:{\:V}_{2}$	$\:{\:\:\:\:V}_{5}$	$\:{V}_{7}$	$\:{V}_{1}$
4	$\:{V}_{5}$	$\:{V}_{7}$	$\:{\:V}_{3}$	$\:{\:\:\:\:V}_{6}$	$\:{V}_{0}$	$\:{V}_{2}$
5	$\:{V}_{6}$	$\:{V}_{0}$	$\:{\:V}_{4}$	$\:{\:\:\:\:V}_{1}$	$\:{V}_{7}$	$\:{V}_{3}$
6	$\:{V}_{1}$	$\:{V}_{0}$	$\:\:{V}_{5}$	$\:{\:\:\:\:V}_{2}$	$\:{V}_{0}$	$\:{V}_{4}$

#### The speed controller (PI)

**Fig. 14:**
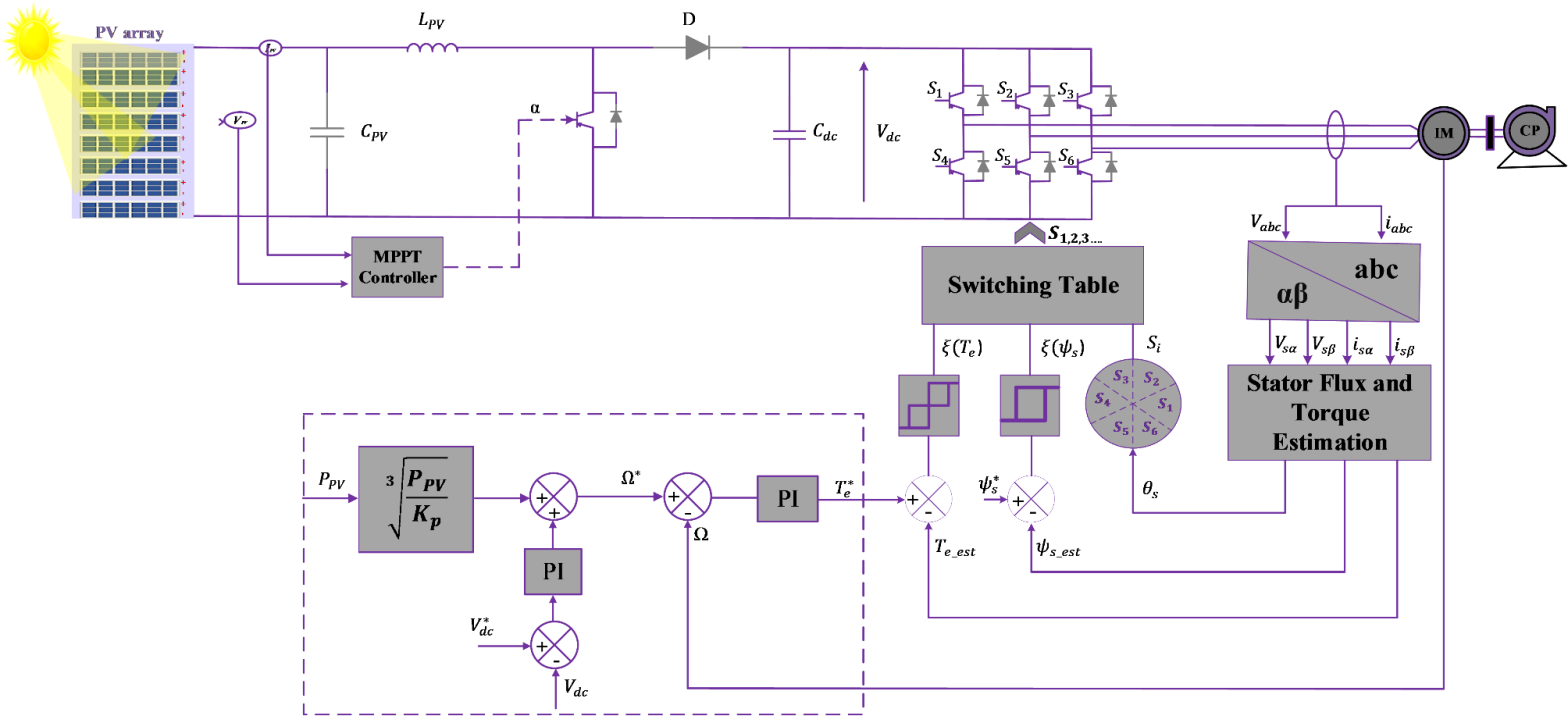
DTC configuration based on the induction motor for PVWPs.

Figure 14 presents the DTC configuration based on the induction motor for PVWPS. Since the PVWPS operates at variable speeds, the speed controller is essential to regulate the reference torque, ensuring efficient system performance. The speed reference $\:{\varOmega\:}_{ref}$ is determined based on two components $\:{\varOmega\:}_{1}$ and $\:{\varOmega\:}_{2}$ as expressed in Eq. 28:

28 $$\:{\varOmega\:}_{ref}={\varOmega\:}_{1}+{\varOmega\:}_{2}$$

These two components address different aspects of the system: The first component $\:\left({\varOmega\:}_{1}\right)$ ​ is proportional to the PV power, which changes with solar irradiance. This relationship, shown in Eq. 29, allows the system to adjust its speed based on available power, ensuring optimal performance.

29 $$\:{\varOmega\:}_{1}=\sqrt[3]{\frac{{P}_{pv}}{{K}_{pump}}}$$

The second component ($\:{\varOmega\:}_{2})$is derived from the output of the DC link voltage PI controller. This controller keeps the DC bus voltage stable, which is important for the system’s performance. The expression of the $\:{\varOmega\:}_{2}$ is given by Eq. 30:

30 $$\:{\varOmega\:}_{2}={\varOmega\:}_{2}\left(n-1\right)+{K}_{PdC}\left({\varDelta\:V}_{dc}\left(n\right)-{\varDelta\:V}_{dc}\left(n-1\right)\right)+\:{K}_{idc}{\varDelta\:V}_{dc}\left(n\right)$$

where $\:{\varOmega\:}_{2}\left(n-1\right)$ is the previous value of $\:{\varOmega\:}_{2}$ ,$\:{K}_{PdC}$ is the proportional gain of the PI controller, $\:{K}_{idc}$ is the integral gain of the PI controller, $\:{\varDelta\:V}_{dc}$ is the difference between the reference bus voltage $\:{V}_{dc}^{\text{*}}$ and the measured bus voltage $\:{V}_{dc}$ ,as described in Eq. 31:

31 $$\:{\varDelta\:V}_{dc}={V}_{dc}^{\text{*}}-{V}_{dc}$$

### DTC based on artificial neural network controller (ANN-DTC)

Torque and flux ripples present significant challenges in DTC, as none of the switching vectors in the inverter capacity to supply the required input voltage for achieving the desired changes in torque and flux^41^. Therefore, to enhance the performance of the studied system by decreasing flux and torque ripples and increasing average efficiency, this section proposes a DTC based on an ANN. In this approach, the ANN controllers replace the speed controller, switching tables, and hysteresis comparators of the Conventional DTC. The basics of the ANN-DTC for IM are shown in Fig. 6.

#### Data collection for the ANN-DTC

**Fig. 15:**
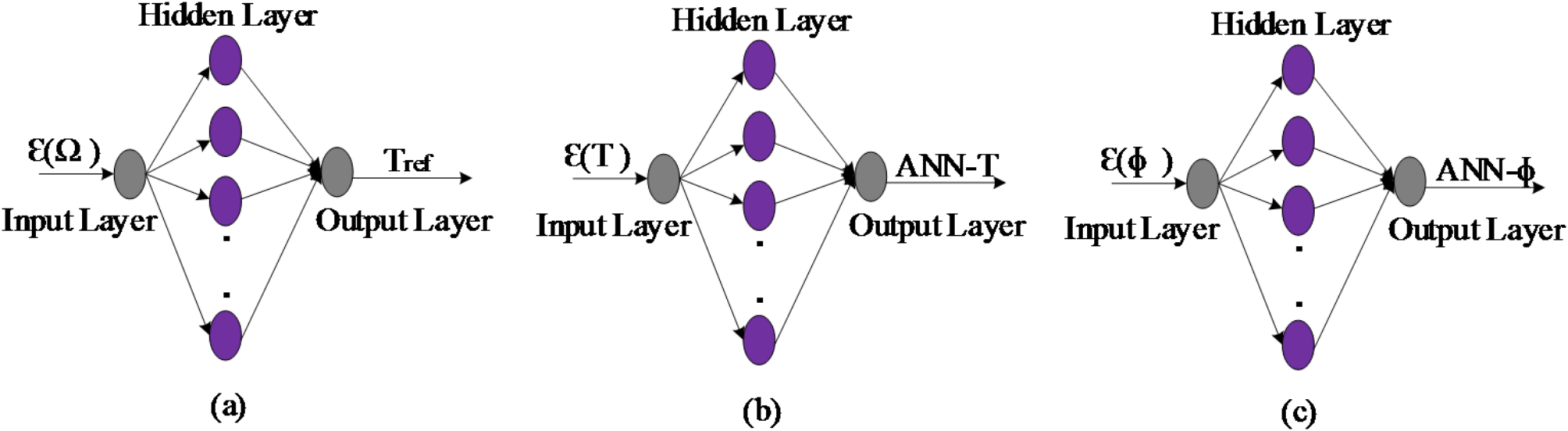
Modeling of feed-forward ANNs for speed controllers (**a**), torque controller (**b**), and stator flux controllers (**c**).

**Fig. 16:**
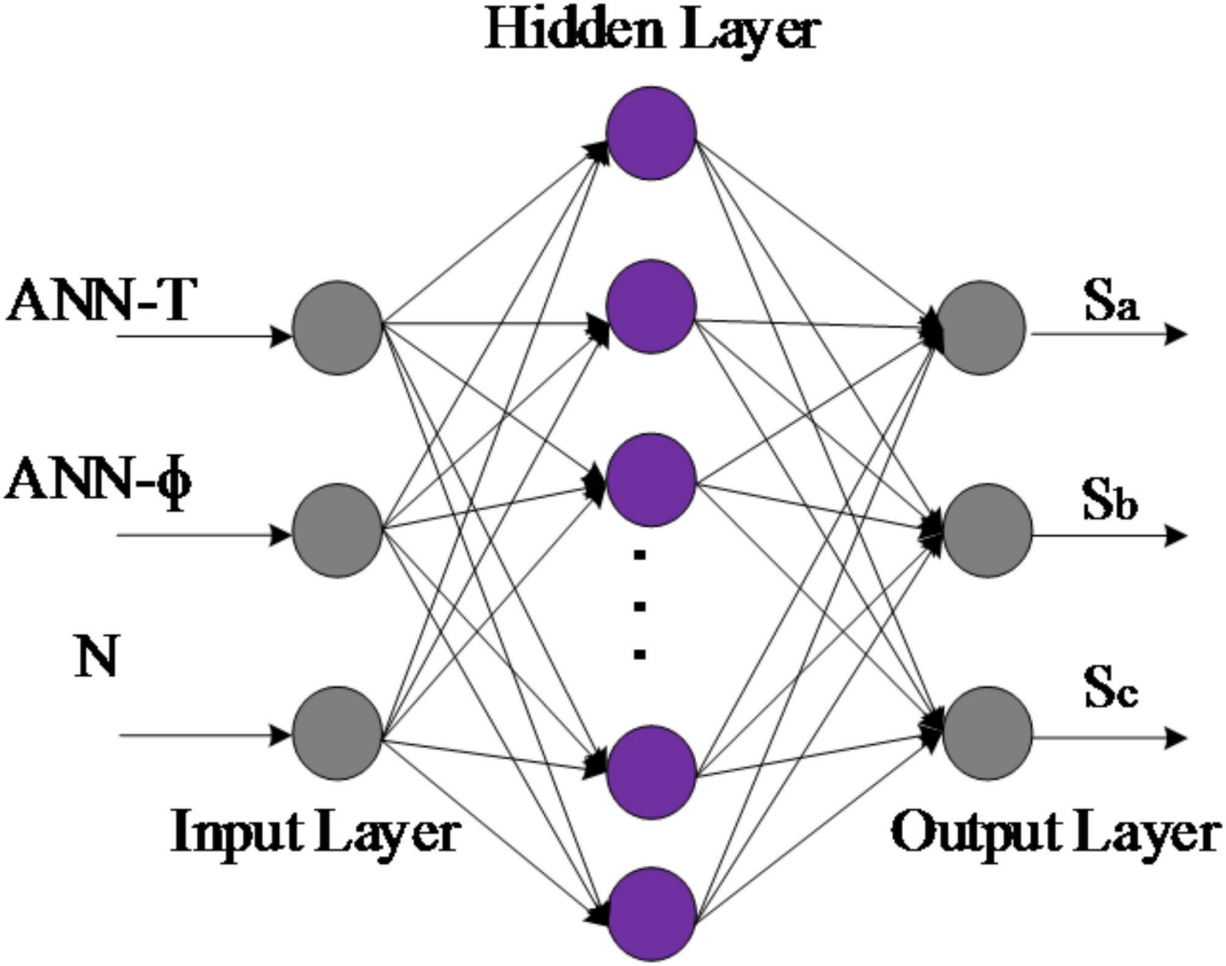
Modeling of feed-forward ANNs for switching tables applied to the stator of the IM.

The proposed DTC-ANN consists of four ANN controllers. The first one replaces the conventional PI speed controller. The input of this ANN controller is the speed error, which represents the difference between the reference speed and the measured speed. The controller’s output is the torque reference, as depicted in Fig. 15a. The second and third ANN controllers replace the two hysteresis comparators, as displayed in Fig. 15b,c, with inputs being the stator flux and torque errors. The outputs of these ANN controllers, along with the sector number, are applied as inputs to the last ANN controller, which replaces the switching table, as illustrated in Fig. 16. The output of the switching table neurons is used to generate the inverter’s on-off switching pattern. The input-output data patterns for training were generated through MATLAB/Simulink simulations using the DTC control simulation outcomes. These outcomes, which include variables such as stator flux, torque, and motor speed, were loaded from the workspace to create the dataset necessary for training the ANN-based DTC controller. As mentioned in the previous section, the number of hidden layers and neurons for speed, torque, flux, and switching table controller is determined using a trial-and-error method. The activation functions for the hidden layer neurons are calculated using the tangent sigmoid function (tansig in MATLAB), while linear functions are used for the output (purelin).

#### Network training for the ANN-DTC

**Fig. 17:**
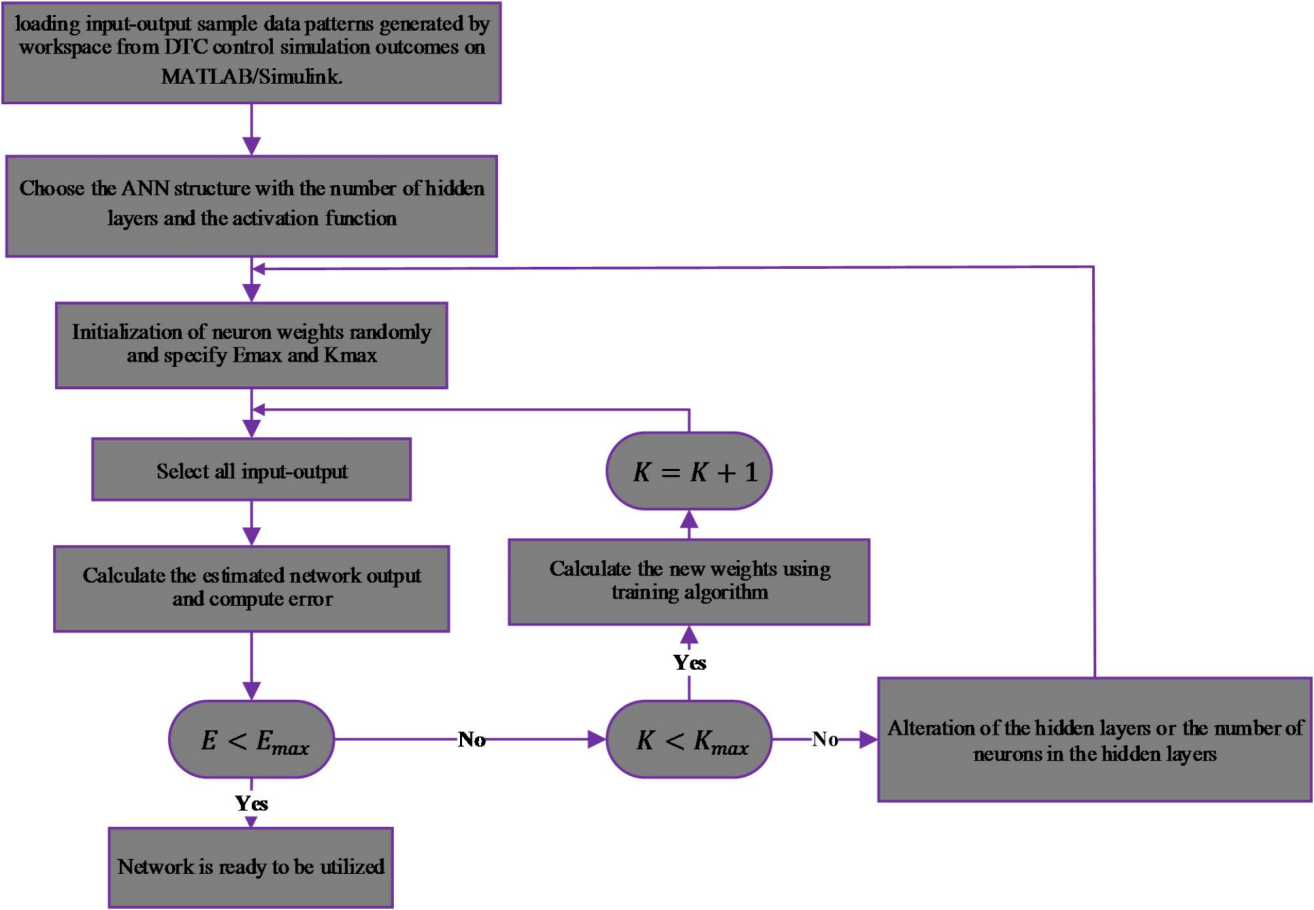
Flowchart of a backpropagation learning algorithm for building neural networks.

The ANN controllers are trained using MATLAB nntraintool. During this training, the neural network adapts its weights and biases to reduce the error between its predicted and actual output. This optimization is achieved using feedforward networks and the back-propagation Levenberg-Marquardt algorithm. The concrete backpropagation training procedure is depicted in the flowchart presented in Fig. 17. The ANN-DTC training text is repeated multiple times, and the best results are obtained from several trials by modifying the parameters for each ANN controller applied to the IM. The neural network’s parameters are shown in Table 3.

**Table 3:** Parameters of ANN-DTC.

	ANN-$\:{\Phi}_{s}$	ANN-T	ANN-Ω	ANN-ST
Training parameters	Feed-forward			
Activation function of Hidden layer	Sigmoid			
Activation function of the Output layer	Linear			
Back-propagation algorithm	Levenberg‒Marquardt			
Number of the input neurons	1	1	1	3
Number of hidden neurons	19	19	10	10
Number of output neurons	1	1	1	3
Number of epochs	1000	1000	1000	1000
Learning rate	0.5			

**Fig. 18:**
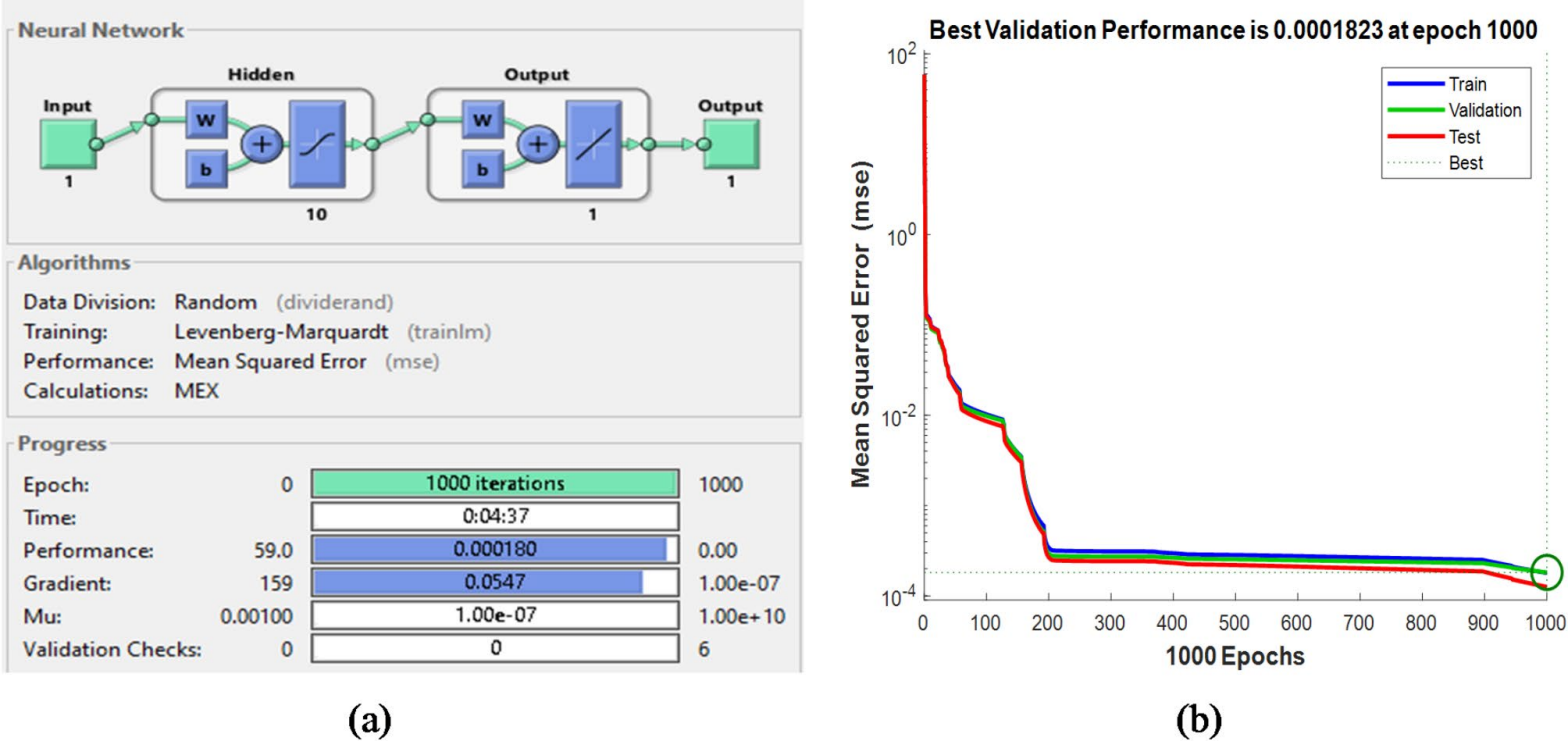
(**a**) Structure of the speed controller based on ANN and its training evolution (**b**) Mean Square Error Performance.

**Fig. 19:**
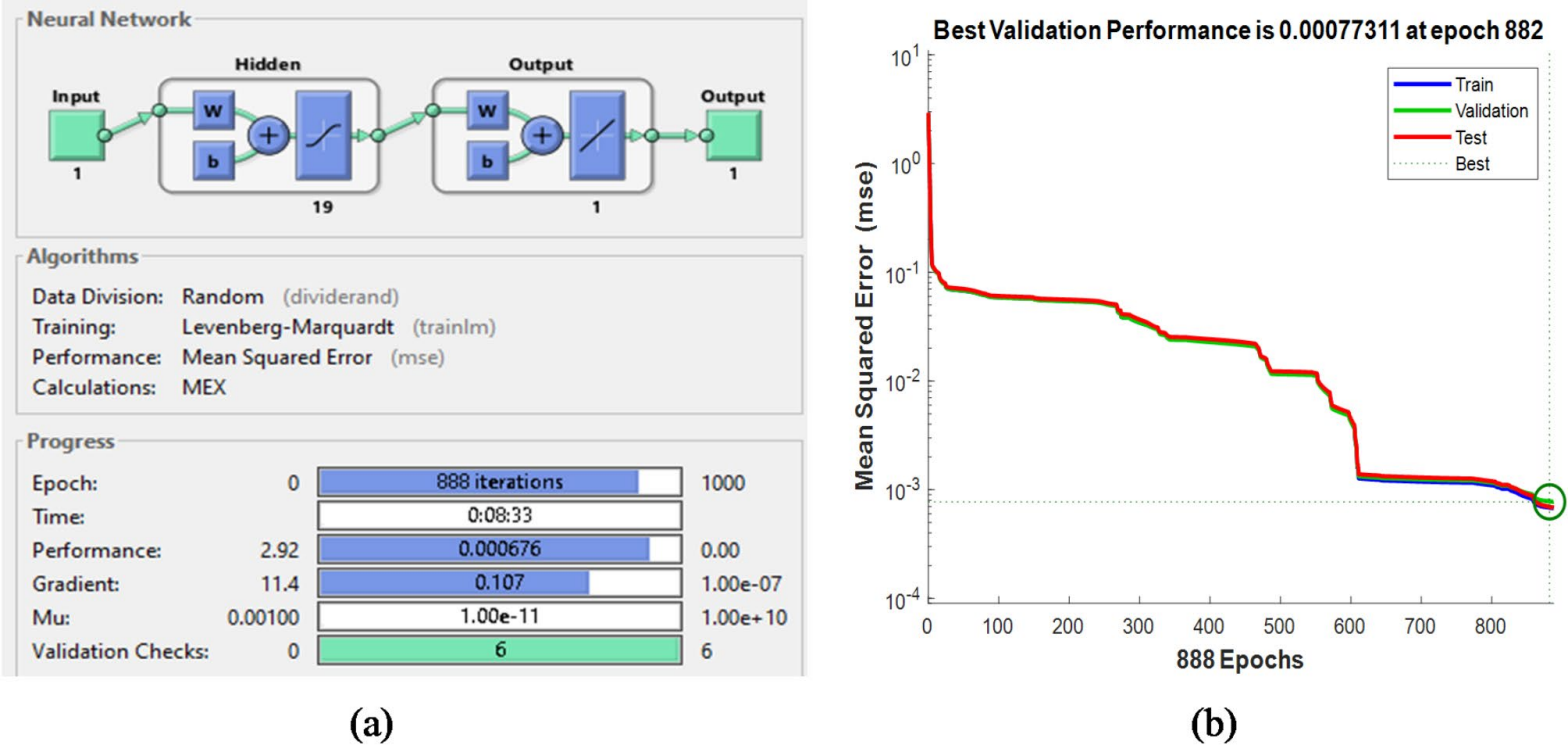
(**a**) Structure of torque controller based on ANN and its training evolution (**b**) Mean Square Error Performance.

**Fig. 20:**
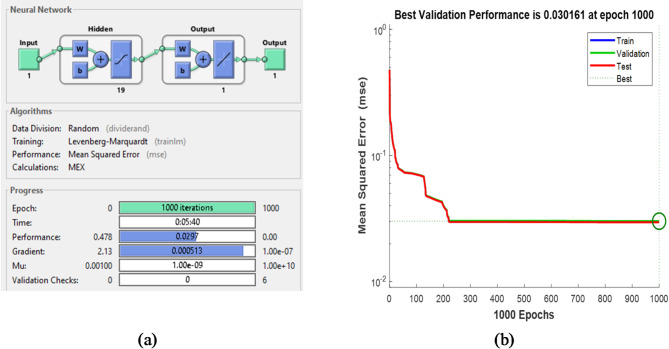
(**a**) Structure of flux controller based on ANN and its training evolution (**b**) Mean Square Error Performance.

**Fig. 21:**
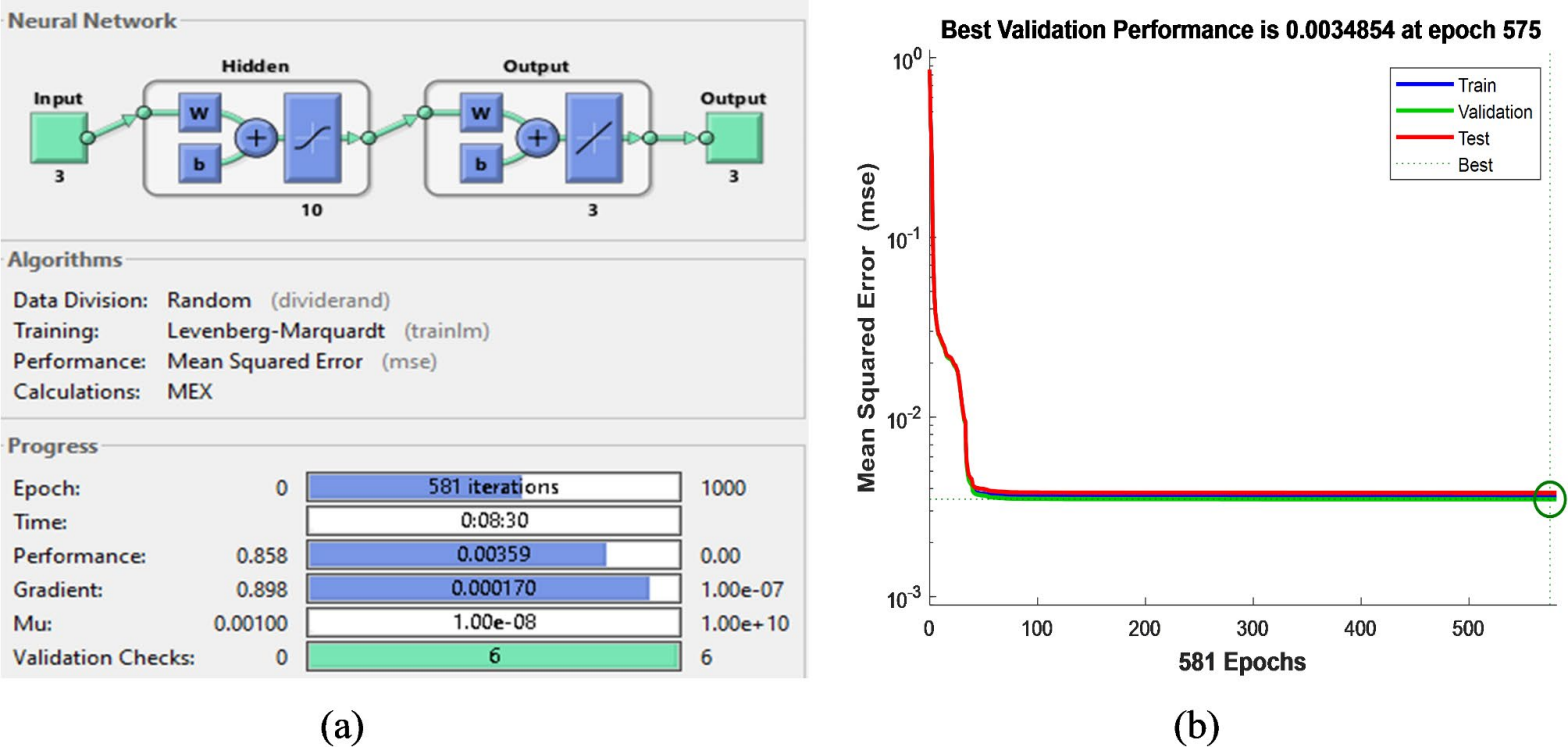
(**a**) Structure of switching table based on ANN and its training evolution (**b**) Mean Square Error Performance.

The training results for each ANN control block are displayed in figures from Figs. 18, 19, 20 and 21, where Figs. 18a, 19a and 20a, and 21a illustrate the architectures of 1-10-1-1,1-19-1-1,1-19-1-1 and 3-10-3-3 for speed, torque, stator flux, and switching table, respectively. Figures 18b, 19b and 20b, and 21b show the evolution of the MSE over time, which decreases to 1.82 × 10^−4^ and 3.01 × 10^−2^ at 1000 epochs for the speed and flux controllers, respectively, 7.73 × 10^−4^ at 882 epochs for the torque controller, and 3.48 × 10^−3^ at 575 epochs for the switching table controller.

## Simulation results and interpretation

In this section, a simulation study is conducted using the MATLAB/Simulink environment to compare the performance of the PVWPS based on the proposed ANN-DTC technique on the one hand and the DTC technique on the other hand. The maximum power point is achieved by employing the proposed ANN-MPPT. Tables A1, A2, A3, and A4 show the parameters required for the PV panels, the boost converter, the DTC controller, and the IM, respectively.

### Performance of the studied system at the startup

**Fig. 22:**
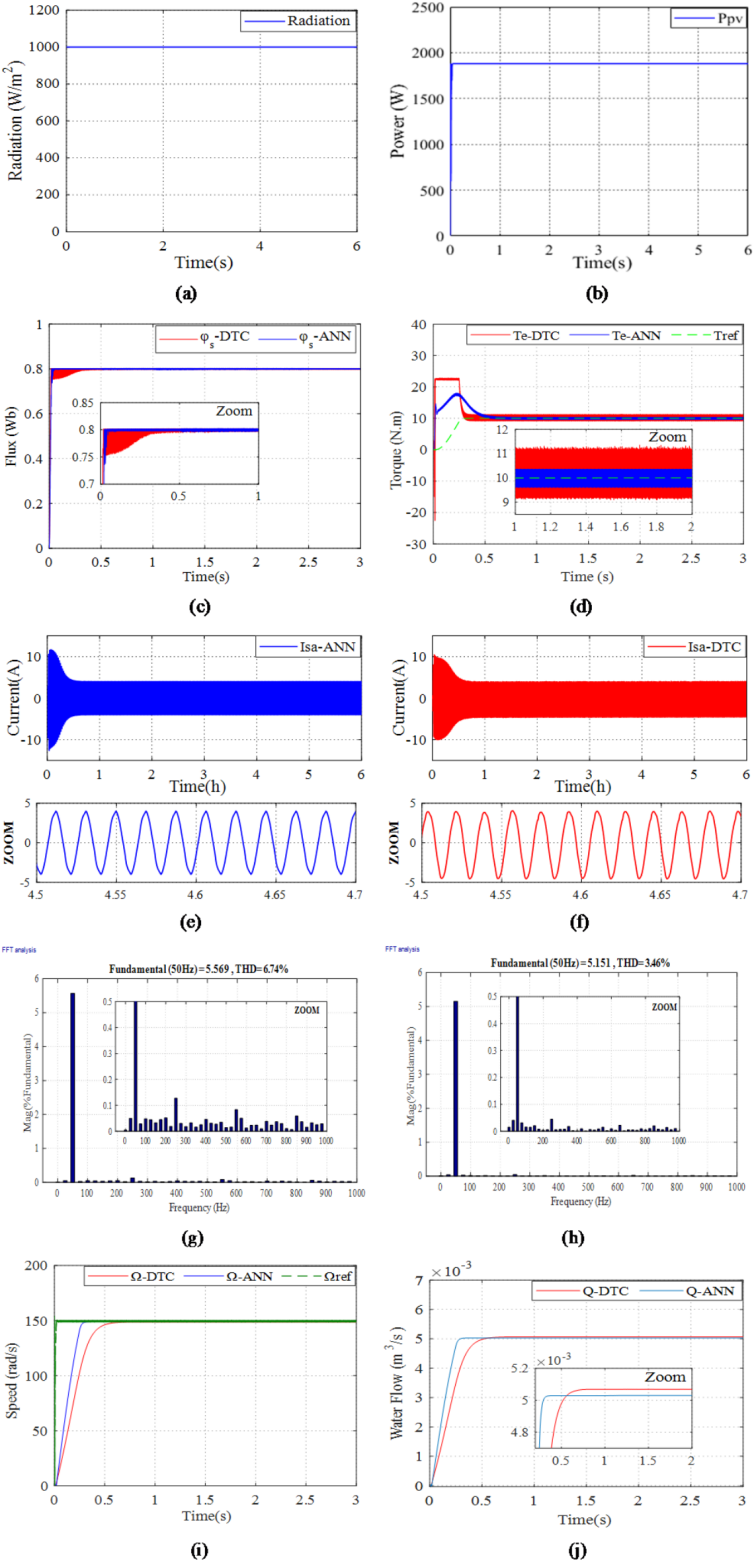
The results of PVWPS under constant radiation: (**a**) fixed radiation, (**b**) PV power produced, (**c**) ANN-DTC and DTC Stator fluxes, (**d**) ANN-DTC and DTC electromagnetic torque, (**e**) the stator current using ANN-DTC, (**f**) the stator current using DTC, (**g**) the THD for stator phase current using DTC (**h**) the THD for stator phase current using ANN-DTC, (**i**) ANN-DTC and DTC Rotor speeds, (**j**) ANN-DTC and DTC water flow.

The first simulation is performed to evaluate the efficacy of the studied system with the suggested controllers under constant radiation of 1000 W/m^2^ as depicted in Fig. 22a.

Figure 22b displays the power generated by the photovoltaic system. It demonstrates that under normal conditions with a radiation level of 1000 W/m^2^, the PV power exhibits a rapid and consistent increase until it reaches its peak of 1880 W at 0.01 s, which is sufficient to effectively operate a 1500 W motor. This showcases the strength of the ANN-MPPT in accurately tracking and optimizing power generation.

Figure 22c displays the flux vectors of the stator. This figure shows that stator flux reaches the desired reference flux level at 0.8 Wb. Furthermore, the figure demonstrates that when the studied system is controlled using DTC, the stator flux exhibits higher fluctuations compared to ANN-DTC, with values of 0.05 Wb and 0.01 Wb, respectively. This indicates an 80% improvement.

Figure 22d presents the electromagnetic torque. This figure illustrates that for irradiation of 1000 W/m^2^, the overshoot of the electromagnetic torque reaches 18 Nm for ANN-DTC and 22 Nm for DTC until 0.4 s. Subsequently, it stabilizes and maintains a reference value of 10 Nm. Moreover, during the stabilization period, as shown in the zoom-in, the torque ripples using ANN-DTC are lower compared to DTC, with values of 0.84 Nm and 2.22 Nm, respectively, showcasing a significant improvement of 62.16%.

Figure 22e,f illustrate the stator current using DTC and ANN-DTC, respectively, with zooming in. Based on zooming in, it can be observed that distortions in the case of ANN-DTC (Fig. 21e) are reduced compared to DTC (Fig. 21f).

Figure 22g,h show the total harmonic distortion (THD) of the current for the DTC and the ANN-DTC, respectively, with zooming in. It is observed that the THD produced by the DTC is 6.64%, while the THD achieved with the ANN-DTC is reduced to 3.46%, representing an improvement of 47.89%.

Figure 22i,j illustrate the rotor speed and water flow, respectively. Based on Fig. 22g, when employing ANN-DTC, the rotor speed quickly converges to its reference value at 0.1 s with minimal fluctuations and high precision. In contrast, with DTC, the rotor speed reaches its reference value at 0.7s and displays significant fluctuations around it. It can be seen from Fig. 22h that the pumped water volume obtained from the studied system using ANN-DTC is higher which reached 5 * 10 ^− 3^than that obtained using DTC which reached 4.90 * 10^− 3^.

### The studied system’s performance under daily profile radiation

**Fig. 23:**
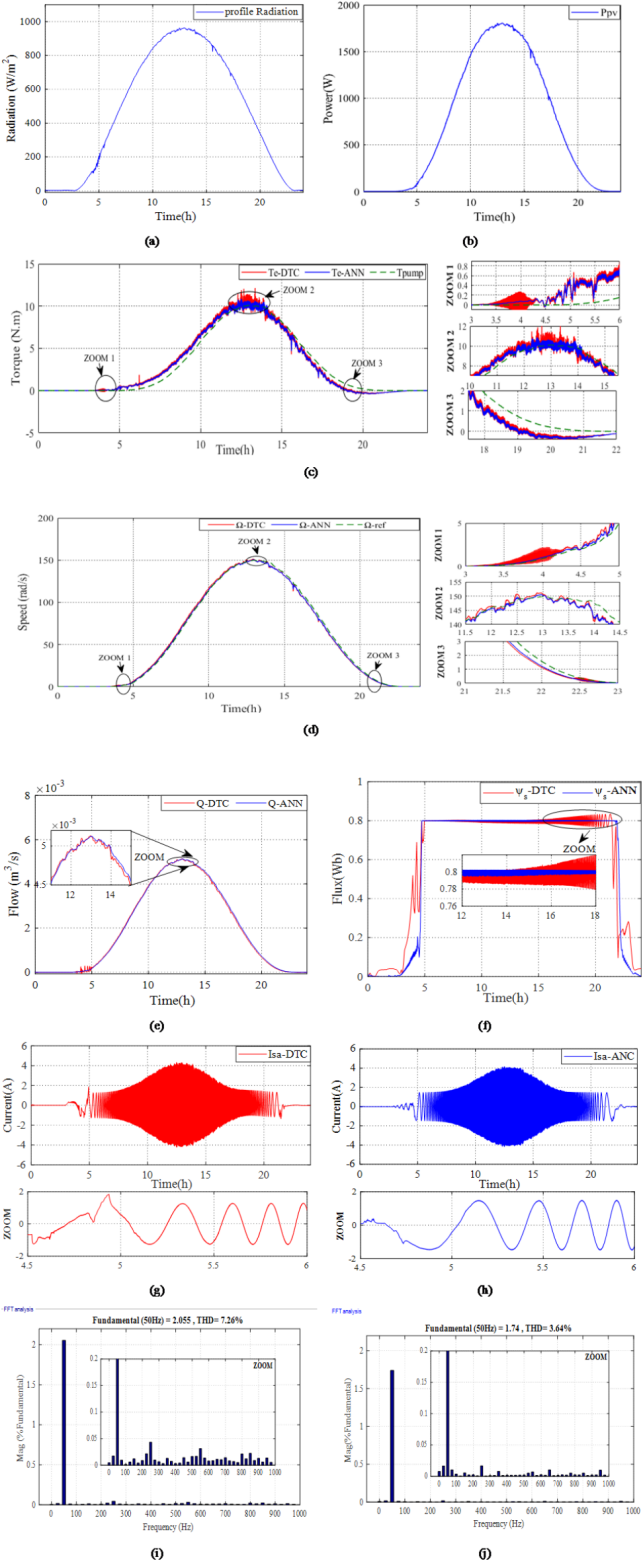
The results of the PVWPS under daily profile radiation: (**a**) Daily Profile radiation, (**b**) PV power, (**c**) ANN-DTC and DTC electromagnetic torque, (**d**) ANN-DTC and DTC Rotor speeds, (**e**) ANN-DTC and DTC water flow, (**f**) ANN-DTC and DTC stator flux, (**g**) the stator current using DTC, (**h**) the stator current using ANN-DTC, (**i**) the THD for stator phase current using DTC (**j**) the THD for stator phase current using ANN-DTC.

The second simulation is conducted to assess the efficacy of the suggested controllers for the studied system, using a real profile during the month of June (2020) in Fes City, Morocco. Figure 23a illustrates the progression of the daily profile of radiation.

Figure 23b shows the PV power. According to this figure, the PV power changes throughout the day in response to the varying levels of radiation. It starts with low or zero before sunrise, then increases as the radiation rises during sunrise, reaches its peak at midday, when radiation is at its highest, and then decreases as solar radiation decreases during sunset. Furthermore, this figure also illustrates the rapid and accurate increase in power throughout the day, reaching the MPP with precision and robustness, thanks to the implementation of the proposed ANN-MPPT algorithm.

Figure 23c shows the electromagnetic torque response during the daily profile of radiation for the studied system using the suggested ANN-DTC and the conventional DTC. Based on the obtained results, the proposed ANN-DTC results in fewer ripples with a value of 0.09Nm. By minimizing these ripples, the studied system can operate more smoothly and efficiently, resulting in improved performance and reliability. In contrast, the conventional DTC approach exhibits high levels of ripples with a value of 0.4Nm.

Figure 23d,e depict the rotor speed and water flow, respectively, for the studied system using both DTC and the proposed ANN-DTC. The simulation results in Fig. 23d with zooming in demonstrate that the proposed ANN-DTC surpasses the DTC approach in terms of response time by values of 2.5 h and 4.8 h, respectively, which resulted in an improvement of 47.79%. Furthermore, it achieves a significantly higher volume of pumped water under the daily profile, as illustrated in Fig. 22e.

Figure 23f presents the stator flux. It is clear from this figure that the stator flux closely tracks the daily radiation profile. Furthermore, it can be noticed that the stator flux attained using the ANN-DTC approach shows fewer ripples and a more favorable response compared to the conventional DTC method, with values of 0.049 Wb and 0.012 Wb, respectively. This leads to an improvement of 62.16%.

The waveforms of the stator current for employing both DTC and ANN-DTC are presented in Fig. 23g,h. The results, zoomed in from 4.5 h to 6 h demonstrate that the three-phase stator current waveform obtained through DTC (Fig. 23g) deviates from a sinusoidal shape with an important distortion. In contrast, the waveform achieved through ANN-DTC (Fig. 23h) exhibits a sinusoidal nature with low distortions.

Figure 23i,j present the total harmonic distortion (THD) of the current for the DTC and ANN-DTC, respectively, with a zoomed-in view. This figure shows that the THD for the DTC is 7.26%, whereas the ANN-DTC reduces it to 3.64%, achieving an improvement of 49.86%. 

### Summary of the simulation results

**Fig. 24:**
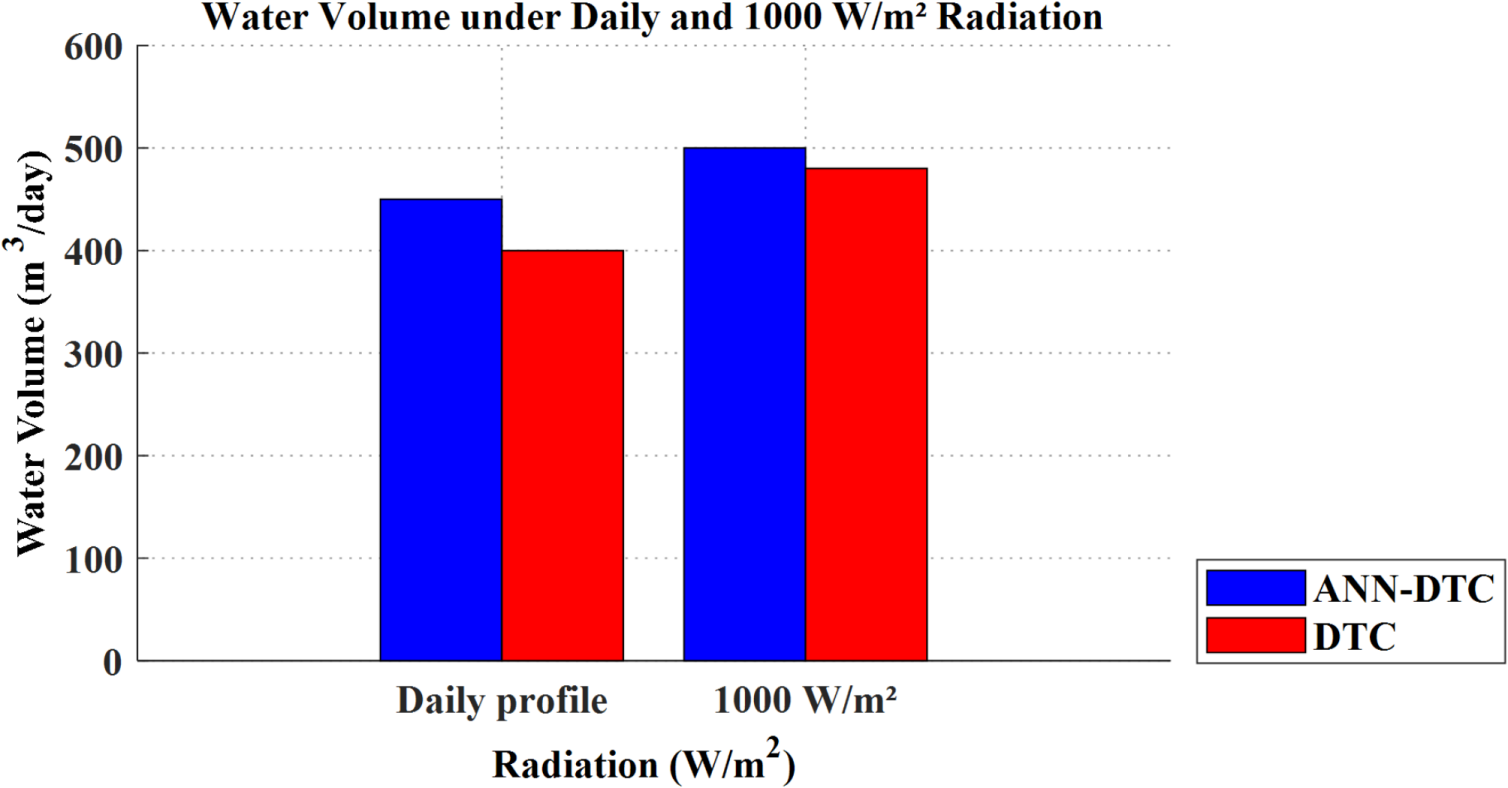
Comparison of water volume between conventional DTC and proposed ANN-DTC under daily profile and constant radiation (1000 W/m^2^).

The performance of the studied PVWPS using the ANN-DTC and the DTC is compared in Table 4; Fig. 24. Table 4 demonstrates the ANN-DTC’s ability to deliver faster response times, significantly lower torque and flux ripples, and reduced THD current. These enhancements, as reflected in Fig. 7, result in a higher pumped water volume under both constant irradiation at 1000 W/m² and the daily profile, displaying the ANN-DTC’s superior efficiency and reliability.

**Table 4:** Performance measures for the conventional DTC and the proposed ANN-DTC.

	Radiation w/m^2^							
	1000				Daily profile			
	Response time (s) of Ω	Ripples (*N* m) of $\:{\:T}_{e}$	Ripples (Wb) of $\:{\phi\:}_{s}$	THD Current (%)	Response time (s) of Ω	Ripples (*N* m) of $\:{\:T}_{e}$	Ripples (Wb) of $\:{\phi\:}_{s}$	THD Current (%)
Conventional DTC	0.7s	2.22	0.05	6.74	4.8 h	0.4	0.04	7.26
Proposed ANN-DTC	0.1s	0.84	0.01	3.46	2.5 h	0.09	0.012	3.64
Improvement	85.71	62.16	80	48.66	47.79	77.5	75.51	49.86

To illustrate the superiority of the proposed ANN-DTC in photovoltaic water pumping systems (PVWPS), a comparative analysis is conducted against several established methods, including Field-Oriented Control (FOC), Direct Torque Control (DTC), and Fuzzy Logic-based DTC (FL-DTC). The findings reveal significant advancements ANN-DTC achieved, particularly in rapid response, minimized torque ripple, and enhanced robustness. As detailed in Table 5, ANN-DTC exhibits the fastest response time at just 0.1 s, outperforming DTC at 0.41 s, FOC at 0.8 s, and FL-DTC at 0.15 s. Additionally, it achieves a remarkable reduction in torque ripple to 0.84 N·m, substantially lower than DTC’s 2.22 N·m and FOC’s 3.2 N·m, while being closely comparable to FL-DTC at 0.95 N·m. Furthermore, the “Very High” robustness rating underscores ANN-DTC’s superior reliability across varying operational conditions compared to DTC and FOC. This comprehensive comparison positions ANN-DTC as the most effective method, particularly excelling in enhancing response time, minimizing torque ripple, and improving system stability within photovoltaic water pumping systems (Table 6).

**Table 5:** Performance comparison of MPPT techniques (T = 25 °C, G = 1000 W/m^2^).

Publication reference	MPPT controllers	Response time(s)	Oscillation (W) power	Efficiency (%)
^1^	P&O	0.013	4.87	93.55
^44^	INC	0.01	2.96	94.6
^16^	KF-MPPT	0.009	0.8	97.7
Proposed technique	ANN-MPPT	0.003	0.4	98.90

**Table 6:** Performance comparison of ANN-DPC with recent control techniques.

Publication reference	Methods	Response time(s)	Torque Ripple (*N* m)	Robustness
^39^	IFOC	0.8	3.2	Low
^43^	DTC	0.41	2.22	Medium
^44^	FL-DTC	0.15	0.95	High
Proposed technique	ANN-DTC	0.1	0.84	Very-high

In addition to the comparison between DTC and ANN-DTC, the performance of the proposed ANN-based MPPT was also evaluated against conventional MPPT techniques, such as Incremental Conductance and Perturb and Observe. Table 5 presents a comparison of these methods based on their response time to achieve maximum power and the degree of power oscillation at T = 25 °C, G = 1000 W/m². The results show that the proposed ANN-based MPPT achieves faster response times and lower power oscillations, demonstrating superior overall system performance.

## Real-time simulation and interpreting

Real-Time simulation and visualization were conducted using a dSPACE DS1104 board to evaluate the system’s performance. This board, developed by the German company dSPACE, is a comprehensive hardware and software platform designed for real-time development and validation of advanced controller algorithms. This enables the seamless transformation of mathematical models created in MATLAB/Simulink into executable files for the prototyping board, eliminating the need for manual coding.

**Fig. 25:**
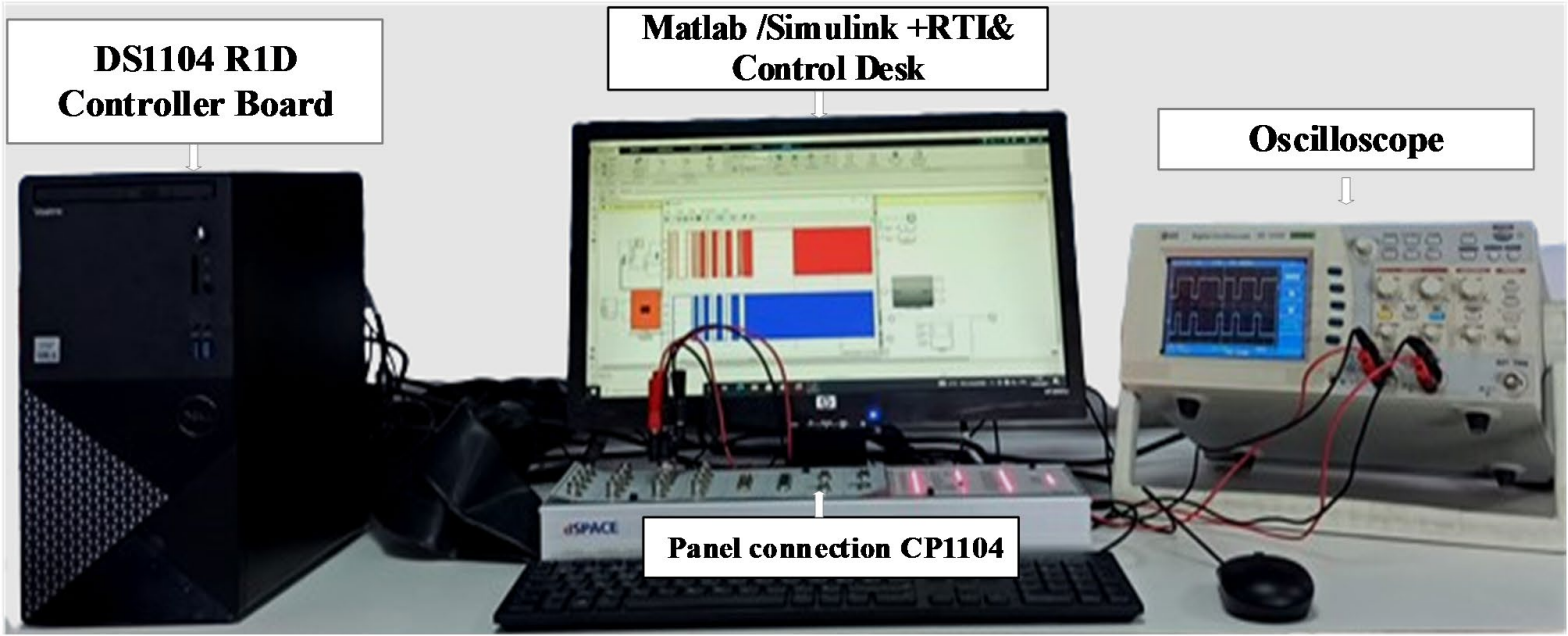
The test bench of the studied system.

As shown in Fig. 25, the DS1104 board is installed inside a computer and connected to MATLAB/Simulink via the Real-Time Interface (RTI) tool, which allows for seamless integration between the software and hardware components of the PVWPS. The RTI tool provides a streamlined environment, enabling the DS1104 board to be directly configured and controlled from within MATLAB/Simulink. Communication between the DS1104 board and the physical elements of the system, such as sensors and converters, is enabled by the CP1104 connection panel. This panel serves as a vital interface for linking key hardware components, ensuring efficient data exchange and control signal transmission. An oscilloscope is connected to the CP1104 panel to capture and display real-time electrical signals like voltage and current waveforms, which are crucial for immediate monitoring and validation of system performance. After developing and compiling the controller model in MATLAB/Simulink using the RTI, an executable code is automatically generated for the DS1104 board. This file is then uploaded to the board via ControlDesk software, which provides a graphical interface for real-time visualization of various system variables.

**Fig. 26:**
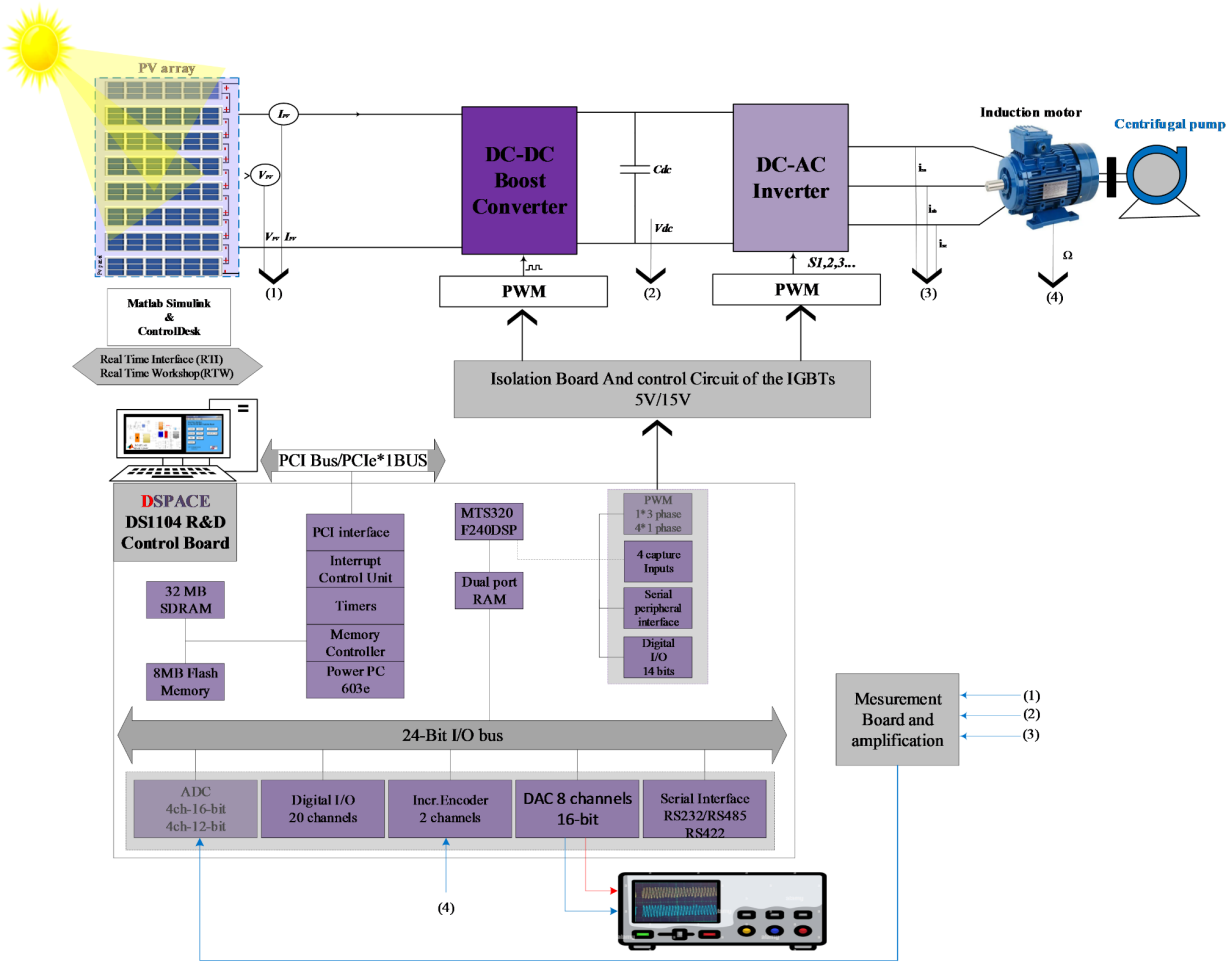
Communication design between DSpace and PVWPS.

Figure 26 illustrates the schematic representation of the connection between the dSPACE board and PVWPS. Validating the proposed PVWPS controllers on the DS1104 board involves these steps:

1. Develop the Controller Model: Build the controller model within the MATLAB/Simulink environment with Real-Time Interface (RTI) integration.
2. Generate Executable File: Translate the Simulink controller model into an executable file specifically designed for the DS1104 board.
3. Upload and Load the Controller: Transfer the generated executable file to the DS1104 board using the ControlDesk software.
4. Real-Time Execution and Visualization: Run the controller algorithm on the DS1104 board in real time and use the ControlDesk interface to visualize the resulting performance data.

**Fig. 27:**
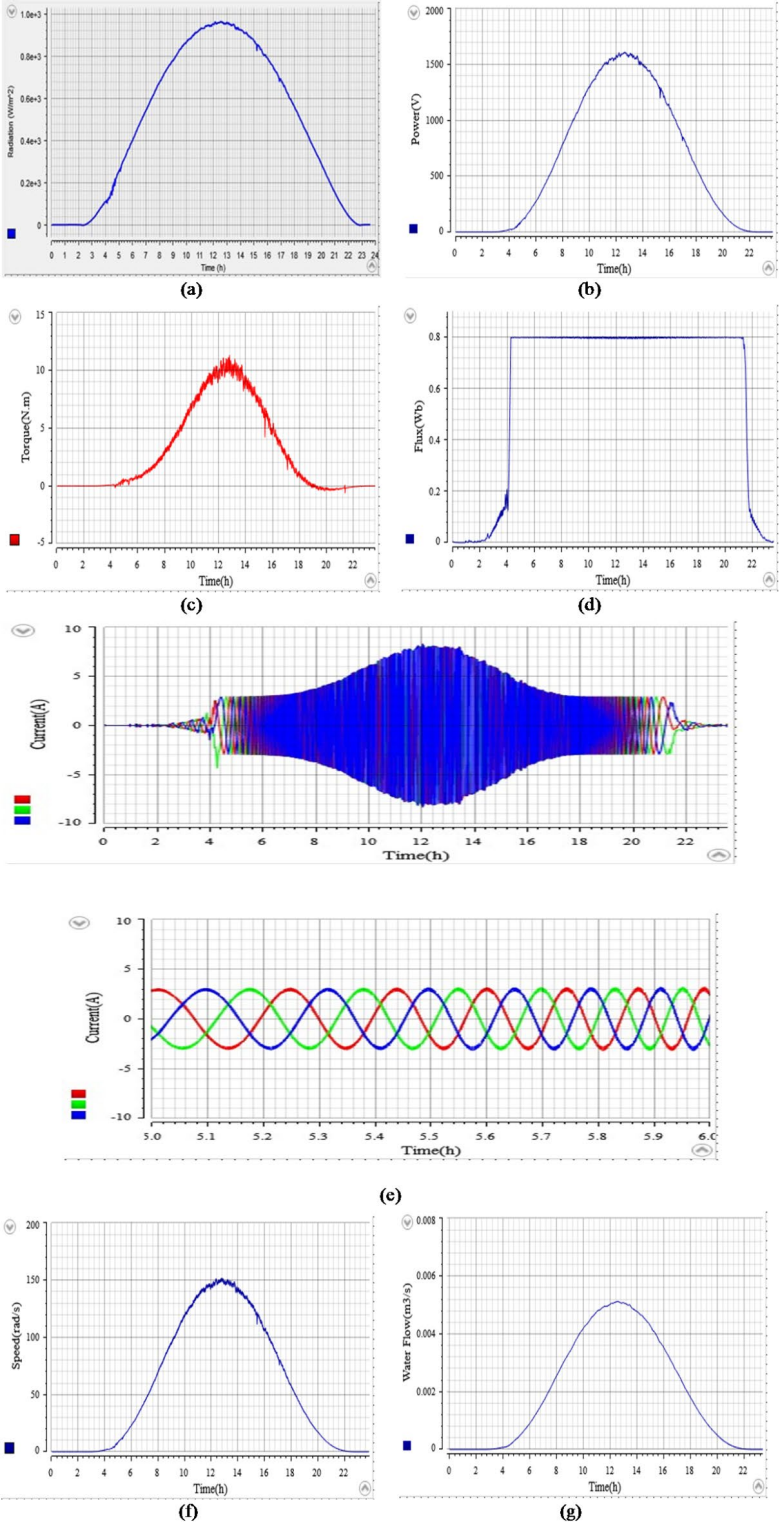
The Real-Time simulation and visualization of the PVWPS under daily profile radiation: (**a**) Daily Profile radiation, (**b**) PV power (**c**) Electromagnetic torque of ANN-DTC, (**d**) Flux response of ANN-DTC, (**e**) the stator current of ANN-DTC and Zooming in of the stator current from 5s at 7 s (**f**) Rotor speeds of ANN-DTC, (**g**) Water Flow of ANN-DTC.

Figure 27a–g show the Real-Time simulation and visualization. Figure 26a presents the daily profile of radiation from the experimental text. Figure 26b presents the practical outcome of PV power. The results show that the experimental findings align with the simulations, demonstrating the excellent performance of the ANN-MPPT in accurately tracking PV power with minimal oscillation near the optimal power point. A small increase in torque ripple is seen in Fig. 27c due to the measurement noise and the machine parameters compared to simulation results. Figure 27d illustrates the experimental findings regarding the stator flux. It is evident that the stator flux closely tracks the daily radiation profile and attains the desired flux of 0.8 Wb, which aligns with the simulation results. Figure 26e presents the three-phase stator current absorbed by the motor. The waveforms exhibit sinusoidal shapes with minimal distortion, indicating that the quality of the experimental waveforms closely resembles those obtained in the simulation. Figure 26f displays the rotor speed. The response time of the speed is reasonable, matching the simulation results obtained in Simulink. Additionally, the speed curve perfectly follows the tracked daily profile of radiation. The Real-Time simulation and visualization of the water flow are displayed in Fig. 26g demonstrates minimal fluctuation and showcases good accuracy, similar to the simulation results. After analyzing both the simulation results obtained from MATLAB/Simulink and Real-Time simulation and visualization gathered from the Controldesk, it has been observed that the studied system exhibits remarkable flexibility and adaptability when subjected to daily profile radiation. This deduction suggests that implementing PVWPS based on ANN controllers would be suitable.

## Conclusion

This paper investigates a standalone photovoltaic water pumping system based on intelligent techniques. The proposed control of the PVWPS studied consists of using an ANN-based MPPT to optimize power extraction from the PV generator under varying solar radiation and ANN-based DTC to control the speed and torque behaviors of the IM. The PVWPS using the DTC and the suggested ANN-DTC were simulated using MATLAB/Simulink and validated with the dSPACE DS1104 board. The results demonstrate significant enhancements in speed, stability, and precision when implementing the proposed ANN-DTC compared to the conventional DTC. These enhancements directly contribute to an increased quantity of pumped water.

The key improvements observed in this study include the following:

- Decreasing torque and flux ripples.
- Improve response time by 85.71% under constant radiation and 44.44% under daily profile radiation.
- Increased quantity of pumped water.

The Real-Time simulation and visualization obtained through ControlDesk confirmed the simulated outcomes obtained from MATLAB/Simulink.

Despite the promising results achieved by the ANN-based MPPT and DTC controllers in simulations and real-time implementation, challenges arose during hardware simulation. These were primarily due to the computational demands of ANN algorithms and their reliance on high-quality, diverse training datasets. Insufficiently representative data limited the controllers’ ability to handle variations like abrupt changes in solar radiation or dynamic loads, reducing reliability. Additionally, deployment in rural areas faces challenges such as limited technical expertise and maintenance support.

Future work will address these challenges by optimizing the computational efficiency of the ANN algorithms and improving the representativeness of training datasets. An experimental test bench incorporating critical PVWPS components will be developed for iterative testing to refine the controllers and address the observed issues. Additionally, hybrid control strategies combining ANN and traditional methods will be explored to balance cost and performance. Cost-benefit analyses and technician training programs will also be undertaken to support deploying and maintaining ANN-based PVWPS in rural and resource-constrained environments. By addressing these limitations, the proposed system has the potential to evolve into a practical, sustainable, and efficient solution for water management in regions with limited resources.

## Electronic supplementary material

Below is the link to the electronic supplementary material.

Supplementary Material 1

## Data Availability

the work has been validated by a simulation on Matlab & Simulink and also by an experimental validation, if the reviewers want more explanations or verify the accuracy of the simulation please contact: Prof. Badre BOSSOUFIbadre.bossoufi@usmba.ac.ma+212663484013thank you for your understanding.

## Abbreviations

PVWPS: Photovoltaic water pumping systems
IM: Induction motor
VSI: Voltage source inverter
DTC: Direct torque control
R&D: Research and development
MPPT: Maximum power point tracking
ANN-MPPT: Artificial Neuron Network based maximum power point tracking
PV: Photovoltaic
ANN-DTC: Artificial neuron network based direct torque control
ANN: Artificial Neural Network
PWM: Pulse width modulation
MSE: Mean square error
PI: Proportional integral regulator

## List of symbols

$\:q$: The electron charge (1.6 × 10^−19^ C)
$\:Is$: The diode reverse-bias saturation current
a: The ideality factor of the diode
$\:K$: The Boltzmann constant (1.38 × 10^−23^ J/K)
T: The temperature
G: The radiation
$\:Ki$: The temperature coefficient of the short circuit current
$\:Kv$: The temperature of the open circuit voltage
$\:{V}_{dc}$: The input DC voltage
$\:{S}_{s,\:abc}$: The switching states of the inverter
$\:\text{M}$: The mutual inductance
$\:{L}_{s}$ ,$\:{L}_{r}$: The stator and rotor inductances
$\:Rs$,$\:{R}_{r}$: The stator and rotor resistances
$\:{I}_{s\alpha\:}$,$\:{I}_{s\beta\:}$: The stator currents in the (α, β) frame
$\:{I}_{r\alpha\:}$,$\:{I}_{r\beta\:}$: The rotor currents in the (α, β) frame
$\:Tr$: The load torque (Nm)
$\:p$: The number of pairs of poles
$\:{\Omega\:}$: The IM rotor speed (rad/s)
$\:J$: The inertia moment
$\:f$: The viscous friction coefficient
$\:Kp$: The centrifugal pump’s constant
$\:Q$: The pumping flow
$\:H$: The pumping head
$\:N$: The rotation speed
$\:{H}_{j}$: The neuron’s output in the hidden layer
$\:{\:x}_{i}$: The input variable from (1 to n)
$\:{w}_{ij}$: The connection weight between the input variable i and neuron j in the hidden layer
$\:{w}_{jk}$: The connection weight between neuron j in the hidden layer and the output neuron
$\:{b}_{j}$: The bias of neuron j in the hidden layer
$\:{b}_{k}$: The bias of the output neuron
O: The output of the neuron in the output layer
N: The number of input‒output training data
$\:{C}_{i}\left(k\right)$: The target output vector
$\:{O}_{i}\left(k\right)$: The ANN actual output vector switching
$\:{\omega\:}_{n}\left(k+1\right)$: The new weight
$\:{\omega\:}_{n}\left(k\right)$: The old weight
$\:\eta\:$: The learning rate
