# Supplementary material for: Improving photovoltaic water pumping system performance with ANN-based direct torque control using real-time simulation
Source: Sci Rep. 2025 Feb 2;15:4024. doi: 10.1038/s41598-025-88330-8 (PMC11788436; doi:10.1038/s41598-025-88330-8)
Supplement: Supplementary file 1 — Supplementary Material 1 [file 41598_2025_88330_MOESM1_ESM.docx]

APPENDIX A

TABLE A.1 Characteristics of the PV panel T5-SER-235P at STC

| **Characteristic** | **values** |
| --- | --- |
| Maximum power${(P}_{\mathrm{mpp}})$ | 235 W |
| Current at $P_{\mathrm{mpp}}$ ${(I}_{\mathrm{mpp}}$ ) | 7.99A |
| Voltage at$P_{\mathrm{mpp}}{(V}_{\mathrm{mpp}})$ | 29.42V |
| Open circuit voltage ${(V}_{\mathrm{oc}})$ | 36.96V |
| Short circuit current ${(I}_{\mathrm{cc}})$ | 8.48A |

TABLE A.2 Parameter values of the boost converter

| **Component used** | **Expression** | **Used value** |
| --- | --- | --- |
| $\alpha$ | $\alpha=\frac{V_{dc}-V_{mppv}}{V_{dc}}$ | 0.41 |
| $L_{PV}$ | $L_{PV}=\frac{V_{mppv}\alpha}{\Delta If_{s}}$ | 3 mH |
| $C_{dc}$ | $C_{dc}=\frac{6\alpha V_{LL}I_{L}t}{\sqrt{3}(V_{dc}^{*2}-V_{dc}^{2} )}$ | 1172 µF |
| $V_{dc}$ | $V_{dc}=\frac{2\sqrt{2}\times V_{LL}}{\sqrt{3}}$ | 400 V |

TABLE A.3 The parameters of the conventional DTC

| Torque hysteresis controller ($H_{Te})$ | ± 0.02 |
| --- | --- |
| Flux hysteresis controller ($H_{\emptyset s})$ | ± 0.002 |
| Sampling frequency | 10 khz |
| The parameters of the PI speed controller | $k_{p}$=0.96, $k_{i}$=38 |

TABLE A.4 Induction motor parameters

| **Parameters** | **Values** |
| --- | --- |
| Power (P) | 1.5KW |
| Nominal speed(Ω) | 1420 rpm |
| Nominal frequency (f) | 50 Hz |
| Stator resistor (Rs) | 4.850 Ω |
| Rotor resistor (Rr) | 3.805 Ω |
| Stator inductor (Ls) | 0.274 H |
| Rotor inductor (Lr) | 0.274 H |
| Mutual inductor (M) | 0.258 H |
| Pairs of Poles (P) | 2 |
| Rated voltage | 230V |
